# Sustainability in health care by allocating resources effectively (SHARE) 3: examining how resource allocation decisions are made, implemented and evaluated in a local healthcare setting

**DOI:** 10.1186/s12913-017-2207-2

**Published:** 2017-05-09

**Authors:** Claire Harris, Kelly Allen, Cara Waller, Vanessa Brooke

**Affiliations:** 1 School of Public Health and Preventive Medicine, Monash University, Victoria, Australia; 2 Centre for Clinical Effectiveness, Monash Health, Victoria, Australia

**Keywords:** Disinvestment, Decommission, De-adopt, De-list, Environmental scan, Health technology, TCP, Resource allocation, Decision-making, Implementation

## Abstract

**Background:**

This is the third in a series of papers reporting a program of Sustainability in Health care by Allocating Resources Effectively (SHARE) in a local healthcare setting. Leaders in a large Australian health service planned to establish an organisation-wide, systematic, integrated, evidence-based approach to disinvestment. In order to introduce new systems and processes for disinvestment into existing decision-making infrastructure, we aimed to understand where, how and by whom resource allocation decisions were made, implemented and evaluated. We also sought the knowledge and experience of staff regarding previous disinvestment activities.

**Methods:**

Structured interviews, workshops and document analysis were used to collect information from multiple sources in an environmental scan of decision-making systems and processes. Findings were synthesised using a theoretical framework.

**Results:**

Sixty-eight respondents participated in interviews and workshops. Eight components in the process of resource allocation were identified: Governance, Administration, Stakeholder engagement, Resources, Decision-making, Implementation, Evaluation and, where appropriate, Reinvestment of savings. Elements of structure and practice for each component are described and a new framework was developed to capture the relationships between them. A range of decision-makers, decision-making settings, type and scope of decisions, criteria used, and strengths, weaknesses, barriers and enablers are outlined. The term ‘disinvestment’ was not used in health service decision-making. Previous projects that involved removal, reduction or restriction of current practices were driven by quality and safety issues, evidence-based practice or a need to find resource savings and not by initiatives where the primary aim was to disinvest. Measuring resource savings is difficult, in some situations impossible. Savings are often only theoretical as resources released may be utilised immediately by patients waiting for beds, clinic appointments or surgery. Decision-making systems and processes for resource allocation are more complex than assumed in previous studies.

**Conclusion:**

There is a wide range of decision-makers, settings, scope and type of decisions, and criteria used for allocating resources within a single institution. To our knowledge, this is the first paper to report this level of detail and to introduce eight components of the resource allocation process identified within a local health service.

**Electronic supplementary material:**

The online version of this article (doi:10.1186/s12913-017-2207-2) contains supplementary material, which is available to authorized users.

## About SHARE

*This is the third in a series of papers reporting a program of Sustainability in Health care by Allocating Resources Effectively (SHARE). The SHARE Program is an investigation of concepts, opportunities, methods and implications for evidence-based investment and disinvestment in health technologies and clinical practices in a local healthcare setting. The papers in this series are targeted at clinicians, managers, policy makers, health service researchers and implementation scientists working in this context. This paper reports an investigation of decision-making infrastructure in a range of local contexts and ascertains the knowledge and experience of disinvestment in one Australian health service network.*

## Background

The concept of disinvestment has emerged in response to rising healthcare costs, rapidly expanding use of health technologies and increasing awareness of ineffective practices and systemic waste in health services [1–7]. Although there is no clear single definition, disinvestment is generally understood to be removal, reduction or restriction of technologies and clinical practices (TCPs) that are unsafe or of little benefit [8]. Removal indicates complete cessation, reduction is a decrease in current volume or delivery sites, and restriction is narrowing of current indications or eligible populations.

Leaders at Monash Health (previously Southern Health), a large health service network in Melbourne Australia, planned to implement an organisation-wide, systematic, integrated, evidence-based approach to disinvestment. The focus was on how a health service guides, directs and makes decisions at organisational level, in contrast to the decisions of individuals regarding their personal practices. Two early decisions affected the scope and direction of this initiative. Firstly, based on a review of the literature and consultation with local stakeholders, it was agreed that the word ‘disinvestment’ should be avoided due to the negative connotations [9]. Secondly, it was felt that undertaking disinvestment in isolation from other decision-making processes was artificial and potentially counterproductive. Hence the ‘Disinvestment Project’ became the ‘Sustainability in Health care by Allocating Resources Effectively’ (SHARE) Program and investment and disinvestment were considered together in the context of resource allocation.

Information to guide healthcare networks or individual facilities in how they might take a systematic organisation-wide approach to disinvestment is lacking [10–19]. Little is known about how to implement or evaluate effective disinvestment initiatives within a health service or how these activities could be integrated with existing processes of health technology assessment and organisational decision-making [20, 21].

Decisions are made at macro (national, state/provincial and regional), meso (institutional) and micro (individual) levels [22]. Each sector of the health system has a decision-making infrastructure within which individuals or groups make decisions on behalf of the jurisdiction or individual facility. However no clear patterns of types of decisions, or where they are made, have been identified for decisions regarding use of health technologies [23, 24]. Lists of criteria for consideration in prioritisation and decision-making have been published for disinvestment [2, 24–27], resource allocation [28–30] and general decision-making [22] but there is little information on decision-making settings or participants in these processes [23].

**Fig. 1:**
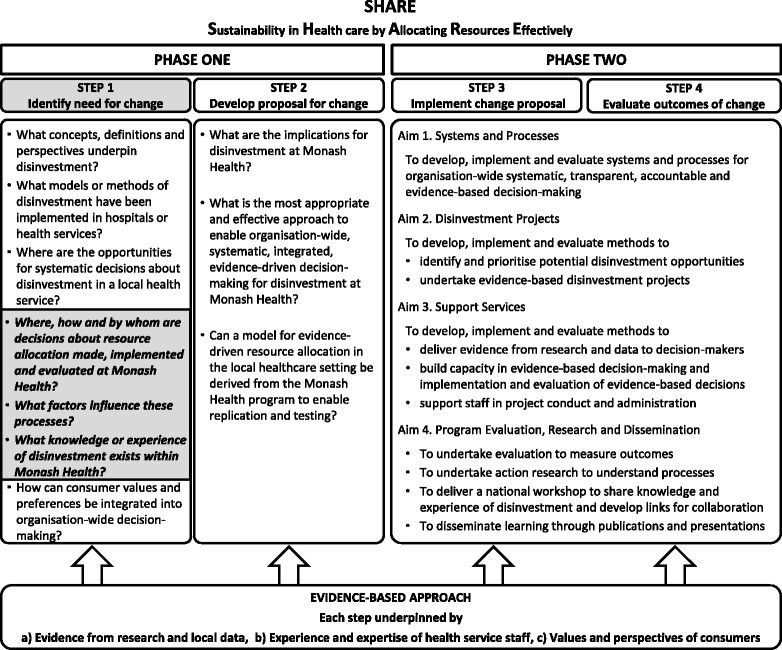
Overview of the SHARE Program

In the absence of guidance from the literature, a two-phased process was proposed to identify and then evaluate potential opportunities for disinvestment at Monash Health (Fig. 1). The aim of Phase One was to understand concepts and practices related to disinvestment and the implications for a local health service and, based on this information, to identify potential settings and methods for decision-making. The aim of Phase Two was to implement and evaluate the proposed methods to determine which were sustainable, effective and appropriate at Monash Health.

Preliminary explorations at Monash Health did not find any decision-making settings that had an existing process to consider disinvestment, hence new systems and processes were required. Two areas with potential had been identified: the mechanisms for spending money, such as purchasing and procurement, and the mechanisms for allocating non-monetary resources through guidelines and protocols [9]. The SHARE Program aimed to integrate new systems and processes into existing infrastructure. While there was a broad understanding of where resource allocation decisions were made at Monash Health, detailed knowledge of how they were made, implemented and evaluated was lacking. This lack of information had to be addressed for this aim to be achieved.

In addition to knowing where and how decisions are made, it would also be helpful to understand and learn from local knowledge and experience of disinvestment. Restricting activities to save money or redirecting resources from one area to another to achieve better clinical or corporate outcomes has always occurred in local health services, but has not previously been called ‘disinvestment’ by healthcare staff. Many staff members could provide insights from previous projects that involved removal, reduction or restriction of resources.

Monash Health is a public network of six acute hospitals, subacute and rehabilitation services, mental health and community health services, and residential aged care [31]. Australian public hospitals operate under a state-allocated activity-based fixed-budget model of financing [32]. Staff are salaried and services are provided free of charge. The SHARE Program was undertaken by the Centre for Clinical Effectiveness (CCE), an in-house resource to facilitate evidence-based practice. An overview of the SHARE Program, a guide to the SHARE publications and further details about Monash Health and CCE are provided in the first paper in this series [33].

### Aims

The aim of this project was to investigate current practice in meso-level decision-making at Monash Health and identify local knowledge and experience of disinvestment.

The aim of this paper is to report and discuss the findings of an environmental scan of organisational infrastructure and mechanisms for resource allocation decisions in a large Australian health service network.

### Research questions

Where, how and by whom are decisions about resource allocation made, implemented and evaluated at Monash Health?

What factors influence resource allocation processes?

What knowledge or experience of disinvestment exists within Monash Health?

## Methods

### Case study

The SHARE papers use a case study approach to address the limited understanding of resource allocation processes in health services, particularly regarding disinvestment [34, 35], and the lack of detailed reporting of implementation of change in the literature [36, 37]. Case studies allow in-depth, multi-faceted explorations of complex issues in their real-life settings [38] and facilitate development of theory and interventions [39]. The case study approach enables examination of the complex behaviours of, and relationships among, actors and agencies; and how those relationships influence change [40]. All three case study approaches are used [41].

1. Descriptive: findings are reported in detail to describe events, processes and outcomes to enable replication when successful and avoidance or adaptation when unsuccessful.
2. Exploratory: literature reviews, surveys, interviews, workshops and consultation with experts are used to explore what is known and identify actual, preferred and ideal practices.
3. Explanatory: theoretical frameworks are used to understand and explain the events, processes and outcomes.

### Environmental scan

An environmental scan involves systematic collection, analysis, interpretation and synthesis of information to enable decision-makers to understand current and potential systems, processes, practices and influences in the internal and/or external environment of their organisation to inform future planning [42–44].

In the SHARE Program, these investigations were undertaken using the SEAchange model for Sustainable, Effective and Appropriate change in health services [45]. Each of the four steps in the model (identifying the need for change, developing a proposal to meet the need, implementing the proposal, and evaluating the extent and impact of the change) is underpinned by the principles of evidence-based practice to ensure that the best available evidence from research and local data, the experience and expertise of health service staff and the values and perspectives of consumers are taken into account. In this context, health service consumers are considered to be patients and other users of health services; parents, guardians or carers of patients; organisations representing consumers’ interests; and members of the public [46].

The two phases of the SHARE Program, the four steps in the SEAchange model and the three research questions addressed in this paper are outlined in Fig. 1.

This environmental scan follows the ‘searching model’ which *“scans broadly and comprehensively in order to determine the true state of affairs”* [47]. The methods are summarised below and provided in detail in Additional file 1.

#### Scanning taxonomy

**Fig. 2:**
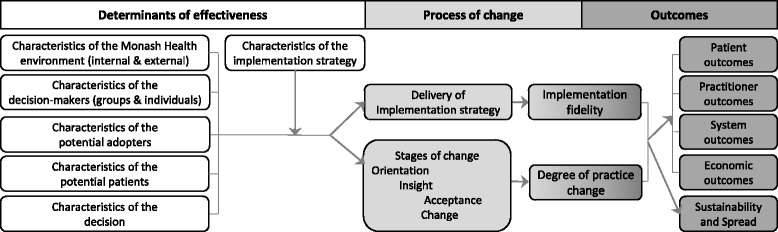
Framework for scanning taxonomy (adapted with permission from Harris et al. [94])

The scanning taxonomy, specified *a priori,* provides a comprehensive set of categories to organise and store information [44]. A theoretical framework for evaluation and explication of implementation of evidence-based innovations was used throughout the SHARE Program to capture and understand the processes and outcomes of change [33]. This was adapted for investigation of decision-making by designating the ‘innovation’ as the decision, the ‘organisation’ as the decision-maker (group or individual) and the ‘external environment’ as the environment in which the decision-maker is situated, in this case Monash Health and the wider environment (Fig. 2). These are equivalent to the task, industry and macro environments described in scanning methodology [44].

#### Scope and sampling

Information regarding the process of allocation of monetary and non-monetary resources for use of TCPs was obtained from interviews, workshops and document analysis.

Purposive, convenience and snowball sampling methods were used, alone or in combination.

Participants were selected to

- cover a wide range of decisions including purchase of capital equipment and clinical consumables; introduction of TCPs in diagnostic and treatment settings; development and/or approval of local protocols and guidelines; implementation of services, programs and models of care; and allocation of staff and organisational capacity in clinics, operating rooms and other facilities; and elicit knowledge and previous experience of disinvestment
- include a range of executives, managers, clinicians and consumers
- represent multiple health professional groups, campuses and clinical specialties

A full description of participants and selection criteria are provided in Additional file 1: Table A.

### Data collection

#### Interviews

Interviews were conducted with representatives of committees and Approved Purchasing Units, managers within a clinical program, and staff with experience in disinvestment projects.

An interview schedule based on the scanning taxonomy was developed, piloted and refined for the committee and program interviews and adapted for the project interviews (Additional file 1: Table B). A less-detailed version was used for the Approved Purchasing Units. In addition, project staff were also asked about the key messages from their experience and what they would do the same way or do differently in future (Additional file 1: Table C). A draft record of interview was sent to interviewees for clarification, comment and/or amendment as required.

#### Workshops

Three structured workshops were conducted; two with the SHARE Steering Committee and one with clinical decision-makers in a large diagnostic service.

The SHARE Steering Committee workshops were based on the first two steps in the SEAchange model for evidence-based change [45]. The workshops were run by the project team and included a presentation, structured discussion and completion of worksheets. Details of the presentations, structured discussions and worksheet tasks are included in Additional file 1: Table D. Findings and decisions were documented in minutes and verified by participants at the next meeting.

The diagnostic service workshop was developed and delivered by an experienced facilitator with no involvement in the SHARE Program. Participants were asked to describe the ideal process for purchasing large capital equipment. Five domains were identified *a priori* and responses on ‘sticky notes’ collected using the nominal group technique were collated under these headings. This method was repeated to identify gaps between the ideal process and current practice. Participants also prioritised key areas for improvement. Notes regarding additional discussions were recorded by project team members. A workshop report was produced and participants were invited to comment.

#### Document analysis

Documents that guided decision-making and/or implementation of resource allocation decisions were sought to provide evidence of the stated positions and methods of administration of the systems and processes at Monash Health and the Victorian Department of Human Services. Documents were identified by key informants and searches within the Policy and Procedure database. Data extraction was based on the scanning taxonomy.

### Data analysis

The three steps for data analysis in environmental scans are 1) organisation of the data using categories determined *a priori*, 2) determination of strengths and weaknesses, and 3) identification of emergent themes [44, 48].

Organisation of data and determination of strengths and weaknesses were undertaken using directed content analysis [49]. Findings were collated and organised in MS Word and Excel based on the scanning taxonomy for the interviews and document analysis, and the domains specified in the workshop activities. Strengths and weaknesses were classified by the project team based on the nature of the item and/or the sentiment expressed by the respondents, and then tabulated using the scanning taxonomy.

Emergent themes were identified using framework analysis [50].

### Synthesis and interpretation

Using the emergent themes, a new framework was developed to provide context for study findings, explain observations, and make the findings meaningful and generalisable. A framework denotes a structure, overview, outline, system or plan consisting of various descriptive categories and the relationships between them [51]. The purpose of a framework is to provide a frame of reference, organise and focus thinking and assist interpretation. Frameworks are descriptive, tend to be high-level and can apply to a wide variety of situations [52, 53].

#### Development of the new framework

1. Identifying concepts and the relationships between them.
  The principles of framework analysis were applied [50].
  Familiarisation occurred during organisation of the data.
  Identification of emergent themes was undertaken in preparation of individual reports for each activity which were used for project decision-making and planning.
  Indexing and charting of all responses within the emergent themes was undertaken when combining these reports to address the research questions, confirming the concepts within the new framework.
  Mapping and interpretation identified the relationships between the concepts.
2. Identifying existing theoretical frameworks that support the new propositions.
  A theoretical approach to decision-making within a health service had been proposed by Williams et al. [54]. The findings from the interviews, workshops and document analysis provided additional detail and increased the scope of the existing approach. The two were combined to form the new framework.
3. Developing a visual representation.
  The concepts and the relationships between them were depicted diagrammatically as the new framework.

This framework was subsequently used to synthesise, interpret and present the findings of the environmental scan in the context of the individual components of the resource allocation process, the elements within each component, and the strengths and weaknesses in the current system.

## Results

Sixty-eight respondents, representing all health professional groups in a range of decision-making contexts across multiple campuses and clinical areas, participated. Representatives of 13 committees; managers of five Approved Purchasing Units; nine Program, Department and Unit Heads; and representatives of 10 disinvestment projects were interviewed and 13 members of the Steering Committee and 18 clinical managers from one department attended workshops. Full details of participants, including response rates and representativeness of samples, are provided in Additional file 1: Table A. Some participated more than once if they had multiple roles; for example as a committee chair responding to interview questions about their role in group decision-making and as a clinical department head participating in a workshop from the perspective of their role as an individual decision-maker. The interviews and workshops were seeking different information, hence individuals were not asked the same questions more than once.

Data collected from these activities informed a range of research questions. Findings related to research questions not addressed in this paper are reported in other SHARE publications [46, 55–58].

Documents analysed from the state government included Victorian Government Purchasing Guidelines, Medical Equipment Asset Management Framework, Targeted Equipment Replacement Program and Health Purchasing Victoria Product Management Guidelines. Documents from Monash Health included the Purchasing Policy, Purchasing Policy Guidelines, Authority Delegation Schedule, Code of Conduct, Conflict of Interest Protocol, Guidelines for management of Gifts and Benefits, Terms of Reference for a range of committees, Application forms, Business case templates, Requisition forms and checklists.

### Framework for the process of resource allocation in a local health service

#### Concepts

Multiple themes emerged from the data and it became clear that Monash Health staff considered decision-making to be only one of several factors in the resource allocation process. These themes can be viewed as the components of the new framework.

Eight components, including Decision-making, were identified. A program for resource allocation requires a Governance component for oversight, direction and control; an Administration component for management and delivery of activities; Stakeholder engagement to ensure that decisions are underpinned by appropriate knowledge and perspectives; and sufficient appropriate Resources to enable the activities. After a decision is made, Implementation and Evaluation components are required to complete the task. In some cases, Reinvestment of savings can be undertaken.

#### Relationships

**Fig. 3:**
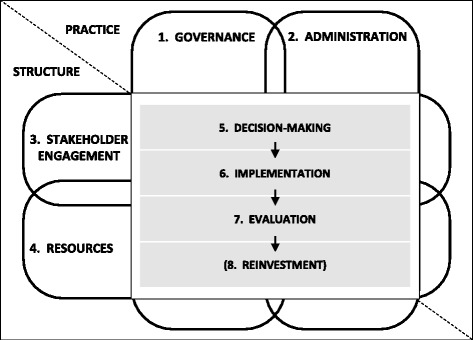
Framework for the process of resource allocation in a local health service

The framework in Fig. 3 presents the relationships between the components. Decision-making, Implementation, Evaluation and Reinvestment (when appropriate) are sequential steps. These four components, plus Stakeholder engagement and Resources, require Governance and Administration. Similarly these four components, plus Governance and Administration, require Stakeholder engagement and Resources. Each component has influence and impact on all of the other components.

#### Building on existing theory

The theoretical approach reported by Williams and colleagues describes the ‘structure’ and ‘practice’ of decision-making in health services [54]. The ‘structure’ elements are based on allocation of people and resources to ‘tasks’ and include committees and their membership, coordination of these elements, and reporting relationships. The ‘practice’ elements include rules and procedures, information gathering and processing, decision processes, performance standards and review.

The findings from Monash Health augment this description by confirming the original elements reported by Williams et al., identifying additional elements and elucidating relationships between them. When combined with the new findings, this theoretical approach, previously focused only on decision-making, can be expanded to a framework considering the whole ‘task’ of resource allocation.

Structure can be described in more detail as ‘who’ and ‘what’ and includes people, systems, policies, requirements, relationships and coordination. Practice addresses ‘how’ through processes, procedures, rules, methods, criteria and customs. There are elements of structure and practice within each of the eight components; these are outlined in Table 1.

**Table 1:** Structure and practice elements of components of organisational decision-making for resource allocation

COMPONENTS	STRUCTURE (Who, What)	PRACTICE (How)
1. Governance	▪ Overseers ▪ Policies for decision-making ▪ Transparency and accountability in all structures ▪ Requirements for addressing conflict of interest^a^ ▪ *Requirements for monitoring, evaluation and improvement of systems and processes* ^*b*^ ▪ *Requirements for reporting*	▪ Oversight ▪ Procedures, guidelines, protocols for decision-making ▪ Transparency and accountability in all practices ▪ Methods of addressing conflict of interest ▪ Methods of monitoring, evaluation and improvement of systems and processes ▪ Methods of reporting
2. Administration	▪ Administrators ▪ Requirements for administration ▪ Relationships and coordination ▪ Communication	▪ Methods of administration, coordination, communication and collaboration
3. Stakeholder engagement	▪ Clinicians, Managers, Consumers, Technical experts, Funders, other relevant parties ▪ Requirements for stakeholder engagement	▪ Methods of identification, recruitment and engagement
4. Resources	▪ Funding sources ▪ Allocation of staff ▪ Access to experts or ways to gain expertise ▪ Information sources ▪ Requirements for resources	▪ Provision of appropriate and adequate funding, time, skills/training, information ▪ Utilisation of resources
5. Decision-making	▪ Decision-makers − Clinicians − Authorised individuals − Authorised groups ▪ Scope of decisions ▪ Type of decisions ▪ Requirements for decision-making	▪ Methods of decision-making − Identification of need/application − Decision criteria − Ascertainment and use of evidence − Reminders and prompts to consider disinvestment − Deliberative process − Documentation and dissemination
6. Implementation	▪ Purchasers ▪ Requirements for purchasing	▪ Methods of purchasing
	▪ Policy and guidance developers ▪ *Requirements for policies and guidance documents*	▪ Methods of policy and guidance development
	▪ Implementers ▪ *Requirements for implementation*	▪ Methods of project management ▪ Methods of change management
7. Evaluation	▪ Evaluators ▪ *Requirements for evaluation* ▪ Type and source of data collected	▪ Methods of evaluation
8. (Reinvestment)	*▪* Requirements for reinvestment/reallocation	*▪* Methods of reinvestment/reallocation

^a^Requirement is used in the sense of performance stipulated in accordance with policies, regulations, standards or similar rules or obligations

^b^Items in italics were not specified by respondents but have been added for consistency across all components

‘Ideal’ elements and ‘actual’ practice at Monash Health for each of the components of the resource allocation process were identified from the responses. The ‘ideal’ elements of structure and practice are represented using the same format for each of the eight components (Table 1). The need for requirements to address conflict of interest, administration, stakeholder engagement, resources, decision-making and reinvestment were reported by respondents, however they did not specifically mention the need for requirements related to other aspects of governance, implementation or evaluation; these have been added for consistency and are noted in italics. The term ‘requirement’ is used in the sense of performance stipulated in accordance with policies, regulations, standards or similar rules or obligations.

### Where, how and by whom are decisions about resource allocation made, implemented and evaluated at Monash Health?

The main messages were consistent across all sources and most of the findings were proposed by multiple respondents, usually from multiple settings. When only one group reported certain findings, or when there were differences in responses between groups, this is noted in the text.

Findings are presented in the context of the new framework for the process of resource allocation in a local health service.

#### 1. Governance

The elements of governance are oversight, policies and procedures, transparency and accountability, mechanisms to address conflict of interest, quality improvement of systems and processes, reporting, organisational requirements for these elements and the people who govern the systems and processes (Table 1).

One of the strongest messages from Monash Health respondents was the need for transparency and accountability. These two principles apply to all components of resource allocation, at all levels and to both structure and practice. They are included here as they can be built into the whole program as an element of governance.

There were notable contradictions between respondents in the knowledge and practice of accountability in decision-making. Individuals and members of committees at the top of their respective decision-making hierarchies reported that they had a clear understanding of how the processes worked and many reported that all decision-makers in the organisation had the same understanding that they did. However many individual and group decision-makers lower down the same hierarchies admitted they were unsure of the processes, some who said they were sure gave answers that were inconsistent with each other, and some reported that there were ambiguities and inconsistencies in the systems and processes. Senior decision-makers reported that they were aware of the differences between recommendations, decisions and authorisation and knew *“who did what”*. Members of higher-level committees saw their role as one of guidance and support in response to robust investigation of decision options which they expected to occur at the lower-level ‘decision-making’ committees. In contrast, some lower-level committee members admitted to being confused about the concepts of ‘decision’ and ‘recommendation’; some saw their role as ‘recommending’ a course of action with the ‘decision’ being made by a higher-level committee, the opposite of the senior decision-makers’ perspective. Senior individual decision-makers reported ‘authorising’ the decisions of their subordinates, while lower-level individual decision-makers did not always know who to report a decision to and whether formal authorisation was required.

Monash Health had specific requirements related to governance of resource allocation. These included policies and procedures for decision-making, a requirement for conflict of interest to be included as a standing item on the agendas of relevant committees, and reporting requirements were outlined in committee Terms of Reference. There was less formal governance of individual decision-makers.

There were no requirements for quality improvement in decision-making systems and processes. At the program level it was noted that *“since there was no formal decision-making process, there was no process of review”*.

#### 2. Administration

The elements of administration are relationships, coordination, communication, collaboration, delivery of the administrative activities, organisational requirements for these elements and the people who undertake them (Table 1).

Another strong message from Monash Health respondents was the potential for duplication and gaps in decision-making and implementation due to a lack of coordination, communication and collaboration. Many committee members reported a lack of awareness of the roles and responsibilities of other committees and a lack of referral and other communication processes. Similarly, many project staff noted the need for coordination between projects and better communication of their activities and subsequent findings within the organisation. Individual decision-makers reported that they communicated with colleagues but also noted failure of others to communicate with them.

Other than reporting structures, there were few formal relationships between decision-making groups and individuals. There were no organisational requirements regarding administration of decision-making.

#### 3. Stakeholder engagement

The elements of stakeholder engagement are identification, recruitment and engagement and the organisational requirements for stakeholder involvement (Table 1). Monash Health had no organisational requirements for stakeholder engagement in these settings.

There were many examples within Monash Health of multidisciplinary representation in decision-making groups and efforts to include representatives from departments, units and sites that would be affected by decisions. However there was also a strong message about the current lack of consultation with the relevant clinical groups when decisions were made by managers. Decision-making *“in isolation”* was noted to be a problem in multiple settings and *“fragmentation”* and a *“silo mentality”* were used to describe decisions made without consideration of the areas they will impact upon or consultation with relevant stakeholders. While inclusion of, or consultation with, all internal stakeholders in decision-making processes was widely supported, there were some difficulties in finding adequate staff time to enable this.

In contrast, the Technology/Clinical Practice Committee (TCPC) responsible for oversight of introduction of new technologies and clinical practices (TCPs) was the only group that included consumer representatives [59]. Although there was support for consumer participation, and several committees were either planning to introduce a consumer representative or became interested during the interview process, several others were unsure about the benefits of consumer participation and some thought that because of the nature of the topics they considered it was inappropriate to include consumers.

#### 4. Resources

The elements of resources are funding sources, allocation of staff time, access to experts or ways to gain expertise, information sources such as evidence from research and local data, and organisational requirements for resources (Table 1).

A lack of resources was reported across all the components, but was particularly emphasised in the context of administration of committee functions.

Monash Health had generic support staff such as librarians and business managers, and also allocated staff with expertise in health technology assessment, data analysis and finance to assist decision-makers. Provision of expertise to support applicants was formalised for the TCPC [59], but there were no other organisational requirements for provision of resources.

The distinction between funding for decision-making, implementation and evaluation processes and funding for equipment purchases was acknowledged; but respondents noted two inherent links. Firstly, effective decision-making and prioritisation for large equipment purchases requires strategic coordinated planning which in turn requires the availability, and knowledge of, *“consistent, ongoing, guaranteed capital funding”*. Secondly, availability of ongoing funding and knowledge of potential funding sources is required in the decision-making process as, in addition to the capital costs of purchasing equipment, decision-makers must also consider costs of training and ongoing costs such as consumables. Respondents noted lack of strategic planning, lack of future funding plans and lack of funding for training and consumables as significant barriers to effective decision-making at Monash Health.

#### 5. Decision-making

The elements of decision-making are scope and type of decisions, requirements for and methods of decision-making, and the decision-makers themselves (Table 1). Decision-makers were clearly identified and the scope of their decisions well-documented, however Monash Health had no requirements for any other aspect of decision-making.

##### 5.1 Decision-makers

Although clinical decisions about use of TCPs for individual patients at the micro level have major implications for implementation of disinvestment and other resource allocation decisions made at macro and meso levels [60–62], they were beyond the scope of the SHARE Program which was focused in the meso context. Clinical decisions are included in the overview for completeness but were not investigated in this study (Table 2).

**Table 2:** Decision-makers and scope and types of decisions for resource allocation

DECISION-MAKERS
Clinicians
Health practitioners delivering patient care.
Authorised individuals
Authorised individuals include Board Members, Executive Directors, Directors and Managers at all levels within the organisation. They are designated by their role in the organisation, for example ‘Director of Pharmacy’, rather than as a named individual ‘John Smith’.
Authorised groups
Authorised groups can be classified into those with
▪ ongoing roles and responsibilities for decisions such as the Board, Executive Management Team, Standing Committees, Approved Purchasing Units and Profession-specific groups such as the Nursing Executive.
▪ a specific, often time-limited, purpose such as a project Steering Committee, a Procurement Evaluation Committee to purchase a large piece of equipment and special initiatives like the High Cost Drugs Working Party of the Therapeutics Equivalence Program.
SCOPE OF DECISIONS
Clinicians make decisions for individual patients within the limits of parameters outlined in their position description, relevant professional standards and any local credentialing requirements.
Authorised individuals and groups make decisions on behalf of the organisation which impact on all patients, all staff or identified subgroups.
Individuals are authorised to make decisions on behalf of the organisation within a range of specified parameters outlined in their position description or the Authority Delegation Schedule.
Committees and other groups are authorised to make decisions on behalf of the organisation as stipulated in their Terms of Reference.
Examples of the parameters decision-makers are authorised to work within include, but are not limited to, location (eg South East sites), professional group (eg occupational therapists), specialty area (eg stomal therapy), patient group (eg children), nature of purchase or resource use (eg surgical equipment and consumables) and cost limit (eg up to $10,000).
TYPES OF DECISIONS
Clinical
▪ Clinical decisions arise in the encounter between a health practitioner and an individual patient or client. Their purpose is to assess, treat and/or plan ongoing management of a health issue.
Strategic, operational or professional
▪ Strategic decisions point the organisation in the direction it wants to go; they are captured in strategic goals and policies which reflect a particular position, priority or plan the organisation wishes to communicate to staff, patients and other stakeholders. Strategic planning is usually undertaken at organisation-level driven by the Board, Executive and Senior Managers but can also be undertaken at any level.
▪ Operational decisions make the strategic goals happen; they enable day-to-day operations and are undertaken by managers at all levels.
▪ Professional decisions address standards and methods of practice and are made by senior staff in the discipline to which they are relevant.
Routine, reactive or proactive
▪ Routine decisions are made on a regular basis; examples include annual budget setting processes, monthly committee meetings and reviews of guidelines or protocols at specified intervals after their introduction.
▪ Reactive decisions are made in response to situations as they arise; for example new legislation, product alerts and recalls, critical incidents and applications for new drugs to be included in the formulary.
▪ Proactive decisions are driven by information that was actively sought for this purpose such as accessing newly published research evidence to compare against current practice or interrogating local data to ascertain practices with high costs or high rates of adverse events.
Conditional or unconditional
▪ Conditional decisions specify requirements to be met before or after their implementation; for example availability of funding, clinical indications (eg disease/condition, severity, patient group), authorised practitioners (eg specific training, named individuals), monitoring of outcomes (eg patient outcomes, adverse events, costs), location (eg ICU, Hospital in the Home ), time limitation (eg until 2 year review).
▪ Unconditional decisions have no requirements.
Allocating funds or non-monetary resources
▪ Allocating funds involves spending money or putting it aside to purchase specified items later.
▪ Allocating non-monetary resources can include rostering staff time; specifying health professional groups; providing clinic or operating room time; and developing protocols that direct use of clinical interventions, equipment, drugs, diagnostic tests and referral mechanisms.
Whether to buy or what, where and how to buy
▪ ‘Whether to buy’ is a decision about what is required, for example a new drug to improve patient outcomes, a new scanner to reduce waiting time, consumables for a piece of equipment in current use. These decisions are undertaken by authorised individuals and some of the authorised groups such as Technology/Clinical Practice Committee, Therapeutics Committee, Falls Prevention Committee, etc.
▪ ‘What, where and how to buy’ is a decision about how the requirement is met and considers product and manufacturer reliability, availability of parts and tools, service and maintenance contracts, IT requirements for hardware and software, price negotiations, etc. These decisions are undertaken by the Approved Purchasing Units and groups established for specific purchases.
Purchase of budgeted or unbudgeted items
▪ Decisions to purchase budgeted items are made by the relevant authorised individual, usually the budget holder or their line manager depending on the purchase price and the designated cost limits of their respective approval levels (eg < $10,000, <$50,000).
▪ Decisions to purchase unbudgeted items can only be approved by specified committees and Executive Directors

At the meso level, decisions are made on behalf of the organisation. At Monash Health, the authority to make decisions on behalf of the organisation was delegated to specified individuals and groups (Table 2). Authorised individuals are designated by their role in the organisation and include Board Members, Executive Directors, Directors and Managers at all levels within the organisation. Authorised groups include the Board, Executive Management Team, Standing Committees, Approved Purchasing Units, profession-specific groups such as the Nursing Executive and specific purpose groups such as project working parties.

##### 5.2 Scope and types of decisions

Decisions can be described in a number of ways. The scope and types of resource allocation decisions identified by Monash Health staff are summarised in Table 2.

The scope of decisions that can be made on behalf of the organisation is documented; this is stipulated in position descriptions or the Authority Delegation Schedule for individuals and the Terms of Reference for committees and other groups.

Decisions can be described from many perspectives such as Strategic, operational or professional; Routine, reactive or proactive; Conditional or unconditional; Allocating funds or non-monetary resources; Whether to buy or what, where and how to buy; Purchase of budgeted or unbudgeted items. These are defined in Table 2. A single decision can be more than one type; for example a decision could be ‘reactive’ in response to a critical incident, ‘operational’ as it involves day-to-day management activities, and result in ‘allocation of non-monetary resources’ such as increasing staffing levels in the area of the incident.

##### 5.3 Identification of need/application

Resource allocation decisions in all settings were made reactively in response to situations as they arose. These can be described in three main groups.

- Government or externally mandated change such as new legislation, regulation or standards; national or state initiatives; and product alerts and recalls.
- Clinician or management initiatives arising from awareness of successful projects elsewhere, conference presentations, journals and other publications, and drug and equipment manufacturer promotions.
- Problem solving driven by critical incidents, staff or consumer feedback, changing population needs, changing demand for services and budget shortfalls.

Three committees had application processes; the others did not have formal processes but considered issues brought to the attention of committee members.

Two groups used benchmarking against national, state and local audits in their area of practice as a proactive mechanism to identify a need for change. No other proactive approaches to examining evidence from research or data that might direct, prioritise or inform decision-making were identified.

Disinvestment was not considered as a reason for change per se but activities to remove, reduce or restrict resources were instigated by factors in the three groups above.

##### 5.4 Decision-making criteria

Only one committee (TCPC) and one individual decision-maker used explicitly documented criteria for decision-making. It could be argued that the application forms and business case templates of other committees contained ‘proxy’ criteria, although the decision-makers were not bound to address them all and applicants did not always complete the whole form. Many respondents reported that they had “*mental checklists*” of criteria they usually considered when making decisions regarding allocation of resources. Examples of criteria used in a range of settings are presented in Table 3.

**Table 3:** Examples of criteria for resource allocation decisions

WHETHER TO BUY				WHAT, WHERE AND HOW TO BUY		
Organisation-wide Committee	Program Committee	Department	Individual decision-makers	Approved Purchasing Units	Organisation-wide Committee	Department
Introduction of new health technologies and clinical practices	Purchase of capital equipment	Purchase of capital equipment	Determination of clinical practices and purchase of clinical equipment	General purchasing	Purchase of clinical consumables	Purchase of pharmaceuticals
Explicit criteria required for decision-making	Criteria ‘usually’ considered A weighted ranking is used for prioritisation	Theoretical ‘ideal’ criteria developed in workshop (different to criteria used in current practice)	Criteria ‘usually’ considered	Criteria ‘usually’ considered	Criteria ‘usually’ considered	Criteria ‘usually’ considered
▪ Conflict of interest (Applicant and Committee members) ▪ Evidence of safety, effectiveness and cost-effectiveness (quality of evidence, size of effect and applicability addressed) ▪ Cost ▪ Clinical feasibility (resource implications, training, credentialing and competency assurance addressed) ▪ Access and equity ▪ Legal and ethical implications ▪ Suitable patient information brochure	▪ Equipment serviceability and impact ▪ Clinical risk ▪ Occupational Health and Safety risk ▪ Accreditation and regulatory requirements ▪ Strategic importance to Monash Health ▪ Savings in operational cost and/or ability to generate funds ▪ Improved access	▪ Workload management ▪ Clinical evidence ▪ Patient benefit ▪ Need ▪ Prioritisation of patient groups ▪ Waiting list ▪ Benchmarking ▪ Replacement for obsolescence ▪ Staff capacity ▪ Allocated budget ▪ Ongoing costs ▪ Funding opportunities ▪ Financial benefit to health service ▪ Multi-use of expensive capital ▪ State-wide planning and coordination ▪ Impact on other areas	▪ Quality and safety/clinical risk ▪ Reducing complications ▪ Ease of use ▪ Staff capacity ▪ Cost/cost effectiveness ▪ Consumer demand ▪ Delivery time of machines ▪ Brand changes (implications for spare parts, training, etc.) ▪ Training needs of staff and consumers ▪ Quality of care	All APU purchase decisions are made with commercial/financial consideration including ▪ Price ▪ Cost-effectiveness ▪ Improved supply chain efficiencies Other factors considered ▪ Clinical need ▪ Legal issues including Health Purchasing Victoria contract requirements	▪ Price ▪ Australian standards and regulations for quality and safety ▪ Infection control/ Occupational Health and Safety standards ▪ Serviceability ▪ Business administration such as supply chain and logistics ▪ Meets organisation’s clinical emphasis and infrastructure requirements ▪ Clinical acceptability and effectiveness	▪ Labelling ▪ Quality ▪ Price ▪Pharmaceutical Benefit Scheme status ▪ Acceptance

##### 5.5 Ascertainment and use of evidence

All committees and most individual decision-makers identified evidence from research and local data as key elements needed for decision-making; however only the TCPC required ascertainment of evidence and data in decision-making and explicitly considered the quality and level of evidence used [59]. Research evidence was considered to include assessments of safety, effectiveness or cost-effectiveness. Respondents acknowledged a number of difficulties in accessing and appraising evidence and the frequent lack of evidence for the question being addressed.

Although interviewees were asked whether they used evidence in decision-making and if they assessed the quality of the evidence; these concepts were not defined in the interview process. The responses suggested that their understanding of evidence, evidence-based processes and critical appraisal was not consistent with current research definitions. Respondents did not report using Level 1 research evidence from sources such as systematic reviews or national guidelines. They did not follow any processes to seek the best available evidence. Some mentioned that the committee had *“experts who know the evidence”* and some individuals noted that department heads *“know the research in their areas”*. Therefore, although we have reported that research evidence was used by most decision-makers, we cannot be sure that it was the best, most appropriate evidence for the decision.

##### 5.6 Reminders and prompts to consider disinvestment

The TCPC had an item on the application form asking which current practices could be discontinued when the new TCP was introduced [59]. No other reminders or prompts to consider disinvestment were identified. There was some scepticism about this process: “*It’s all very well to ask the question but it’s very hard to get a clinician to say they will stop doing something*”.

##### 5.7 Deliberative process

Some, but not all, committees required a quorum for decision-making. There was a general sense that committee decisions were achieved through consensus, but many respondents perceived that decisions were often made outside the committee process or were influenced by lobbying. No specific frameworks or methods for deliberation were identified. There were no organisational requirements for these or any other elements of a deliberative process.

##### 5.8 Documentation and dissemination

The TCPC published ‘Decision Summaries’ on the internet and disseminated these through a formal distribution process [59]. One committee did not have any written records of their decisions. The others fell between these extremes, recording minutes or action statements which were not published but could be available on request. The content or quality of documentation was not investigated in this study.

Methods of dissemination included routine meetings, emails, phone calls, memos, clinical handover sessions, education sessions, newsletters (Pharmacy, Chief Executive, Director of Nursing, Medication Safety), nursing communication book, night shift communication book, department website, committee reporting structures, presentations at Grand Rounds, conference papers and posters. Most of these elements were reported by respondents in all settings.

#### 6. Implementation

The elements of implementation are purchasing, guideline and protocol development, practice change, the requirements and methods for these activities and the purchasers, guideline developers and project teams undertaking them (Table 1). Not all elements are required for each decision, for example a purchase may not be involved or a new guidance document may not be required.

All of the information about implementation came from staff undertaking projects mostly initiated within departments. No committees had processes for active implementation of their decisions, some were unclear about whether they were responsible for implementation and others knew they were responsible but had no resources to implement.

##### 6.1 Purchasing

Monash Health mandated ‘separation of function’ where at least two independent individuals or groups were involved in the purchasing process, one to determine whether to buy, the other to determine what, where and how to buy. Only the Approved Purchasing Units could purchase products and services. Examples of the criteria used in purchasing decisions are outlined in Table 3.

This process generally worked well, however lack of communication was also noted between clinicians and managers making decisions to buy and the purchasers enacting them. Having made a decision to purchase, clinicians and managers did not always consider purchasing requirements and often went directly to manufacturers, resulting in either substandard contract outcomes or duplication of effort when it had to be done again. Purchasers assumed that clinicians and managers had considered all the appropriate evidence and other relevant criteria in their decision-making but had no systematic methods to check this, resulting in purchases of potentially inappropriate or ineffective products.

##### 6.2 Policy and guidance development

Some decisions trigger introduction of new, or changes to existing, policies and many, particularly those related to allocation of non-monetary resources, are implemented through local guidelines and protocols. Use of policy and guidance documents was generally accepted and viewed positively.

##### 6.3 Practice change

It was widely acknowledged that projects to implement practice change require skills in project management and change management and that these were generally lacking.

Training and education activities and *“champions”* were routinely used as implementation strategies and were reported to be effective in achieving change and sustainability of the intervention.

#### 7. Evaluation

The elements of evaluation are the type and sources of data collected, requirements and methods of evaluation and the evaluators (Table 1).

Evaluation was highly valued by respondents from all groups, but frequently not undertaken. There were no organisational requirements for evaluation of decisions or projects and only two of the ten projects included evaluation in their project plans. Government funded projects and some committees had their own requirements for evaluation.

Like practice change, it was acknowledged that specific skills were necessary but generally lacking, and lack of resources was reported to be a significant barrier to evaluation.

Analysis of the interview findings identified that there was insufficient information in some of the responses to separate types and sources of data, for example Medication Safety Audits are a source of data but we do not know what types of data were collected using this instrument. Examples of sources of evaluation data used by committees are summarised in Table 4 using the categories from the scanning taxonomy and, where the information is available, details on the types of data collected are also included. In addition to accessing routinely-collected data, some projects collected their own data specific to the project objectives.

**Table 4:** Examples of types and sources of evaluation data used by committees

Process (implementation) and Impact (practice change)
▪ Progress Reports for new TCPs including number of patients treated, number waiting, new referrals (6 monthly)
▪ Medication safety audits (twice yearly)
▪ Continual Review Evaluation through Australian Council of Healthcare Standards Guide (dates in Nursing Strategic Plan)
▪ Established surveillance mechanisms of transfusion practices (ongoing)
▪ Audits of transfusion practice (random, on behalf of Department of Human Services)
▪ Incident reports (as they arise, documented in Riskman software)
Practitioner outcomes
▪ Survey/interview data including user satisfaction and comments (after project implementation)
▪ Clinical practice audits (quarterly)
▪ Incident reports (as they arise, documented in Riskman software)
Patient outcomes
▪ Progress Reports for new TCPs including patient outcomes and adverse events (6 monthly)
▪ Reports of adverse events related to new TCPs (at the time of occurrence)
▪ Infection Control surveillance mechanisms (ongoing)
▪ Incident reports (as they arise, documented in Riskman software)
Economic outcomes
▪ Clinical Information Management databases of routinely-collected data used to assess
− Cost of falls and falls-related injuries (as required)
− Cost of increased length of stay (as required)
− Costs of products (as required)
− Costs of procedures (as required)
System outcomes
▪ Applications for new TCPs including anticipated implications of new TCP on other areas such as intensive care or pharmacy
▪ Reports of 2 year review after introduction of new TCP including actual implications of new TCP on other areas

#### 8. Reinvestment

Reinvestment of resources was viewed as an incentive for disinvestment; however the lack of transparency and consultation in reinvestment of savings was seen as a barrier.

Respondents noted the need for planning for reinvestment. Although the act of reinvestment occurs at the end of the sequence, decisions about whether savings are the primary objective of the process or anticipated as a secondary outcome, how they will be achieved and measured, and where they will be reinvested must occur at the beginning. Reinvestment must be addressed in the decision-making, implementation and evaluation phases if it is to occur.

Respondents reported that resource savings are difficult, in some cases impossible, to measure due to health service accounting practices. Budget-holding cost centres are linked to sites, departments, wards, pharmacy, diagnostic services, operating suites, intensive care units and similar entities. Use of a single health technology or clinical practice involves multiple cost centres and the level of detail required to isolate information within a cost centre for an individual TCP is not available.

Approaches to measuring savings were reported to be too superficial and often did not consider lateral impacts: *“We don’t look far enough for downstream effects; we’re too simplistic in assessment of savings”*. Also *“Cost saving measures in one area can result in increased costs in another area”;* for example a practice change may reduce the length of stay (LOS) but the patients require additional outpatient services. When a project in one department increased costs in another, reallocation of savings to the project department was thought to be unfair.

Financial savings are often theoretical and never become realised. This is particularly evident when the savings are made in bed days, clinic time or operating sessions which are immediately used to treat other patients. Reducing LOS or waiting times for clinic appointments and surgery has considerable benefits, to patients and the health service, but because there are always patients waiting to use the services there are no actual monetary savings. Savings are only realised if the beds, clinics or operating rooms are closed. In addition, the cost per day of a hospital bed is greater at the beginning of an admission than at the end, so reducing the LOS of a group of patients by discharging them a few days earlier is likely to increase total costs if the beds are used for new admissions of higher acuity.

The SHARE Steering Committee was keen to establish and support measurement of savings and methods for reinvestment and proposed flexibility and lateral thinking in development of novel methods and indicators.

### What factors influence resource allocation processes?

The findings are collated and classified using categories from the scanning taxonomy (Fig. 2), to which the components of the resource allocation process (Fig. 3) have been added. Full details are provided in Additional file 2.

#### Strengths and weaknesses

Respondents noted that Monash Health had considerable strengths, but also many opportunities for improvement. One of the main strengths was that decision-makers recognised the weaknesses and wanted to see improvements in transparency and accountability; standardisation of practice; use of explicit decision-making criteria including evidence; stakeholder consultation; information about *“who does what, how the process works and why”;* communication, coordination and collaboration between decision-makers; provision of adequate and appropriate resources; and active implementation and evaluation of outcomes. However there were also notable exceptions; some doctors did not want to be restricted by specified criteria or requirements to find evidence for their decisions and several respondents thought that consumer representation on committees was unnecessary or inappropriate.

#### Barriers and enablers

Interviewees were asked specifically about barriers and enablers that influenced decision-making, implementation and evaluation. Some factors were reported as both a barrier and an enabler; in situations when the factor was present it was reported as a barrier or enabler, and when absent was noted as the reverse. Only the responses received have been recorded, but additional barriers and enablers can be inferred by considering the positive or negative alternatives of those reported.

Many of the barriers and enablers identified by this specific question were also mentioned in response to other questions by interviewees who did not include them in their answer about barriers and enablers. In addition, many other factors that could be considered barriers and enablers emerged from the general responses but not from the specific question. Because of this overlap, separating the factors identified by the specific question about barriers and enablers from the other influencing factors may be a false distinction. To report only the responses to the question about barriers and enablers would not convey all the potential barriers and enablers to resource allocation in this setting, and to add barriers and enablers identified in responses to other questions would require an interpretation from the researchers that may not be appropriate.

Although not synonymous, strengths are aligned with enablers and weaknesses with barriers. The barriers, enablers and other influencing factors have been combined with the strengths and weaknesses in resource allocation at Monash Health (Additional file 2). Specific responses to the barrier and enabler question are identified by italics.

As expected, the well-established generic barriers to effective evidence-based decision-making, implementation and evaluation such as lack of resources, particularly time and skills, lack of evidence and data, clinical autonomy and resistance to change were present at Monash Health, however many new factors specific to resource allocation in the local healthcare setting were identified. Some examples include lack of organisational requirements for rigorous practices in decision-making, implementation or evaluation; lack of support for administration of committees and the high workload involved; perceptions that corporate criteria take preference over evidence of safety, effectiveness and cost-effectiveness: *“what the hospital is concerned about – finances, organisational capacity and risk management – and what the clinician is concerned about – patients”*; difficulty taking off *“clinician hat”* and replacing it with “*manager or decision-maker hat”*; lack of funding for training on new equipment; requirement to buy particular items or brands if they are specified in the state government purchasing catalogue although it is not evidence-based; difficulty measuring and simplistic approach to resource savings; difficulty realising financial savings; and lack of planning and consultation for reinvestment.

#### Differences between medical and nursing decisions

There were notable differences in the decision-making practices of the doctors and nurses interviewed.

There were more levels of accountability and pathways for operational and clinical support and oversight of nursing decisions compared to medical decisions. Nursing staff reported a hierarchy of decision-making and reporting within the program, the site and the organisation. In the clinical program selected, the Medical Program Director gave the medical department heads sole accountability for their decisions as he considered they were the most senior experts in their specialty areas.

Nurses reported making more decisions about changing policies and procedures and fewer decisions regarding large equipment purchases; doctors reported the reverse.

For the individual decision-makers, there was a general feeling among medical interviewees that decisions were made in the best possible way without the use of consistent, explicit, documented criteria and that efforts within the organisation to introduce this encountered resistance. Conversely, some nursing staff welcomed the use of documented criteria for the potential benefits of increasing transparency, standardising practice, decreasing the unintended consequences of some decisions and reducing adverse events.

While research evidence and local data were valued in decision-making for both groups, nursing staff reported the use of local data more often than medical staff. Medical staff noted the use of research evidence in guiding decisions more often than nurses, and also commented on the shortage of research evidence in many of their specialty areas.

### What knowledge or experience of disinvestment exists within Monash Health?

Although the term ‘disinvestment’ was generally unfamiliar, the concept was readily understood by participants. There were multiple settings for explicit and systematic consideration of investment, but no setting was identified that overtly considered disinvestment. Although disinvestment-related decisions to remove, reduce or restrict current practices were undertaken, they were driven by quality and safety proposals, evidence-based practice or a need to find resource savings, and not by initiatives where the primary aim was to disinvest.

Projects involving disinvestment-related activities were easily identified. The ten projects included ranged from small department-level activities to organisation-wide initiatives (Additional file 1: Table A). Most were instigated by department heads and completed within existing departmental budgets.

Interviewees provided a range of reasons for undertaking the projects; these included reducing patient harm, reducing medication error, reducing unnecessary tests, improving communication, standardising care, saving money and saving time. Most projects had more than one of these objectives. Projects were initiated by external mandate, awareness of good practice elsewhere or in response to an internal problem.

Almost all of the responses from project staff regarding implementation would be applicable to any type of change and were not related to the nature of disinvestment. There were only two disinvestment-related references: an expression of frustration arising from the lack of information about how savings were reinvested and an observation that doctors “*don’t care”* about healthcare costs which makes money-saving exercises *“hard to sell”.*

Reflections regarding disinvestment from the committee representatives and individual decision-makers focused on two areas: savings and reinvestment.

## Discussion

### Limitations

The consistency of messages from respondents in a range of professions, positions and decision-making settings provide triangulation for internal validity, however there are some potential limitations to external generalisability and possibility for bias. Only one organisation is represented, and there may be many points of difference with other health services. However many of the findings are similar to research in other decision-making contexts. The details of the ‘where, who and how’ of decision-making will vary between organisations but most of the principles should be the same; individuals and groups will make decisions under certain conditions which can be elucidated for each institution. Selection bias could affect our conclusions if the lack of central documentation of relevant committees and projects prevented ascertainment of all relevant groups or if the single program and department chosen were not representative of their counterparts. It is reassuring that the main messages were consistent across all settings, there were no inconsistencies between groups, but there was some variation within groups suggesting that a range of opinions were captured. To minimise interview bias, records of interview were sent to interviewees and workshop reports were sent to participants. Some interviewees corrected errors or added factual information.

Because this study investigated how decisions were actually made, and sought information from the decision-makers themselves, the lack of consumer participation in the process was reflected in their limited involvement in this study. The only contributions were from the two consumer representatives on the SHARE Steering Committee who attended the workshops. Potential methods and opportunities for consumer engagement in organisational decision-making are explored in Paper 4 in this series [46].

### Contribution of this study

#### Systems and processes for resource allocation

Most of the literature on disinvestment and resource allocation concentrates on the process of making decisions. Although decision-making is a key component of resource allocation, this study highlights seven additional components required for achievement of this task. To our knowledge, this is the first paper reporting this level of detail regarding decision-making settings, decision-makers, scope and type of decisions, strengths and weaknesses, barriers and enablers, and criteria used for allocating resources within a local health service.

#### Decision-makers

In many studies of decision-making, participants are selected from senior positions such as commissioners, board members, Chief Executives, vice presidents, Finance Directors and other executive and senior management roles [10, 23, 30, 63–68]. At Monash Health, resource allocation decisions were not only made by executives and senior managers, they were also delegated to authorised groups and individuals throughout the organisation. It was also clear in this example that senior staff did not always have a full understanding of processes at lower levels.

In previous research, resource allocation has sometimes been considered to be a homogenous process within an institution; for example survey participants at macro and meso level have been asked whether ‘the resource allocation process’ in ‘their organisation’ was fair, whether evidence was considered, or what criteria were used, implying that there was only one decision-making process [10, 63, 64]. However this study found considerable variation in systems and processes within a single health service; criteria varied in nature and scope and ranged from formal documented requirements to “*mental checklists*”; and there were no central sources of information about “*where, who and how*” decisions were made.

These findings suggest that decision-making infrastructure is much more complex than generally portrayed, that there may not be a single way of doing things within large institutions and that we may not be able to generalise from the knowledge and experience of senior respondents.

#### Types of decisions

There are many types of decisions which have not previously been discussed in the literature in this context (Table 2), all of which offer potential to explore and initiate disinvestment.

It is clearly important to investigate decision-making mechanisms for spending on multi-million dollar equipment purchases, however little attention has been paid to decisions that spend millions of dollars on frequently used low-cost items. Millions of cannulae, catheters, dressings and similar consumables are used every year in large facilities. Consideration of safety, effectiveness, cost-effectiveness, ease of use and amount of staff time required in the use of these items provides further disinvestment opportunities and potential for improved outcomes and significant cost saving.

Decisions can be made about spending or saving money, or about allocating non-monetary resources. Most of the research has been on how funds are distributed but decisions that direct the use of drugs, equipment and diagnostic tests; specify health professional groups and referral mechanisms; and allocate staff time and capacity in clinics, operating rooms and other facilities have major impact on resource use. These decisions are made in different settings and by different decision-makers than those making financial decisions and are often implemented through local guidelines and protocols. There are opportunities for systematic consideration of disinvestment in all of these activities [9].

#### Criteria for decisions

Lists of criteria for prioritisation and decision-making at macro, meso and micro levels have been published [2, 22, 24–26, 28–30, 66, 69]. This study illustrates the variation in criteria used by meso-level decision-makers in different contexts within the same institution and the differences in criteria between those deciding ‘whether to buy’ and those deciding ‘what, where and how to buy’ (Table 3).

### Implications for policy and practice

#### Strengths and weaknesses

Monash Health is not unique in the nature or extent of these findings. These issues have also been identified in a range of decision-making contexts [10, 13, 23, 30, 63–68, 70–77]. Current authors reviewing, debating or investigating disinvestment and resource allocation also note similar needs for improvement in decision-making systems and processes [12–14, 16–18, 25, 26, 28, 60, 78–92].

#### Opportunities for disinvestment

Although there were multiple settings for formal and informal decision-making about resource allocation, with the exception of the TCPC application form, none of these expressly considered disinvestment. The current systems were not sufficiently rigorous or standardised to introduce processes for disinvestment, particularly in situations where there was no precedent for using explicit criteria in decision-making. Addressing the limitations in routine decision-making practices would be required as a first step towards evidence-based consideration of disinvestment.

The new framework for resource allocation provides a scaffold on which to build a systematic approach to disinvestment.

The practice elements of the framework provide opportunities to introduce triggers, prompts or even mandatory requirements to consider disinvestment, for example:

- decision-making contexts such as meeting agendas, strategic planning, budgeting, explicit decision-making criteria, application forms, development processes for guidelines and protocols, and authorisation processes
- implementation contexts such as purchase orders, guidelines and protocols, clinical paths, checklists, communication strategies and education programs
- evaluation contexts such as development of performance indicators, audits and reviews

The structural elements within the decision-making component could be used in a similar way, for example:

- decision-makers could be targeted for training to be aware of disinvestment possibilities or provided with examples of successful disinvestment initiatives
- types of decisions could be explored for disinvestment opportunities
- requirements for consideration of disinvestment could be introduced into documents governing scope of decisions such as position descriptions and committee Terms of Reference.

#### Monitoring, evaluation and improvement of systems and processes

Quality improvement in clinical practice and service delivery is well-established and routinely conducted in healthcare facilities. The same cannot be said for quality improvement in organisational decision-making, although it has significant influence on clinical practice and service delivery. All of the components in this framework can be monitored and evaluated and the findings used for improvement.

#### Active implementation and evaluation of decisions

There is a large body of research on decision-making for resource allocation, and a substantial volume of literature on implementation of clinical practice change and evaluation practices, but little on implementation and evaluation of resource allocation decisions. This study demonstrates that it was not uncommon for decisions to be made in our health service without any plans for implementation and, in most cases, not to be evaluated at all. There is considerable opportunity for development of policies and practices for implementation and evaluation of resource allocation decisions.

### Implications for research

Many of the findings from this study are the first of their kind hence, although they provide more information than was previously available, they require confirmation or refutation in subsequent studies.

Investigation of decision-making processes and methods of stakeholder engagement are established fields of research, and some work has been undertaken in the context of disinvestment and resource allocation, however the new information from this study regarding the settings, scope and type of decisions; variation in criteria used; strengths, weaknesses, barriers and enablers; and the opportunities to integrate systematic consideration of disinvestment into the decision-making infrastructure has opened up new research possibilities in these areas.

Methods for guideline development, implementation and evaluation have all been well-researched, but not in the context of resource allocation, and there has been little, if any, investigation of all the other elements of structure and practice in the eight components of the resource allocation process [12, 13, 23, 67]. These are also potential areas for future research.

## Conclusion

Decision-making systems and processes for resource allocation are more complex than previously assumed in many studies. There is a wide range of decision-making settings, decision-makers, scope and type of decisions, and criteria used for allocating resources within a single institution. The level of detail of these and other elements of resource allocation provide opportunities for future research and changes to policy and practice.

## Additional files

Additional file 1: Methods. (PDF 328 kb)

Additional file 2: Strengths and weaknesses, barriers and enablers for resource allocation processes (PDF 463 kb)

## Abbreviations

APU: Approved Purchasing Unit
CCE: Centre for Clinical Effectiveness
LOS: Length of stay
SHARE: Sustainability in health care by allocating resources effectively
TCPC: Technology/Clinical Practice Committee
TCPs: Technologies and clinical practices
