# Supplementary material for: Sustainability in health care by allocating resources effectively (SHARE) 3: examining how resource allocation decisions are made, implemented and evaluated in a local healthcare setting
Source: BMC Health Serv Res. 2017 May 9;17:340. doi: 10.1186/s12913-017-2207-2 (PMC5423420; doi:10.1186/s12913-017-2207-2)
Supplement: Supplementary file 1 — Methods. (PDF 328 kb) [file 12913_2017_2207_MOESM1_ESM.pdf]

The SHARE Program (Sustainability in Health care by Allocating Resources Effectively) 3: Examining how resource allocation decisions are made, implemented and evaluated in a local healthcare setting

Additional File 1

Methods

Contents

Table A. Data collection, analysis and response rates 2
Table B. Interview questions for committee decision-making mapped to scanning taxonomy 5
Table C. Interview questions for previous disinvestment projects mapped to scanning taxonomy 6
Table D. SHARE Steering Committee Workshop Proformas 7
Abbreviations 8
References 8

Table A. Data collection, analysis and response rates

STRUCTURED INTERVIEWS

Staff authorised to make decisions on behalf of the organisation

Aim: To identify and document current processes for making, implementing and evaluating decisions and the factors that influence them.

Inclusion criteria: Staff and consumers authorised to make decisions regarding resource allocation for health technologies and clinical practices at organisation-wide level in group or individual settings.

Sampling: Purposive and snowball sampling was used.

- Twenty-two committees were initially identified from a governance structure diagram. A further 20 were identified through a snowballing method by asking participants in the subsequent interview process, senior managers and Quality Unit staff if they were aware of others. Fourteen of the 42 potential committees met the inclusion criteria (Capital Expenditure, Falls Prevention, Information Systems Governance, Joint Program Quality and Safety, Medication Safety, Operating Suite Product Evaluation, Nurse Standardisation of Practice, Resuscitation, Skin Integrity and Pressure Ulcer, Sterilising Services, Technology and Clinical Practice, Therapeutics and Transfusion Committees and the Executive Management Team).
- Approved Purchasing Units (APUs) have delegated authority from the Board to commit the organisation to a legal and/or financial obligation such as issuing a purchase order or signing a contract. Of the nine APUs, two had been included in the group decision-making committees (Capital Expenditure Committee and Executive Management Team) and five others met the inclusion criteria (Pharmacy, Health Technology Services, Equipment Services, Procurement and Clinical Purchasing, and Materials Management).
- Clinical managers from one clinical program selected for its high use of health technologies were identified from the program's intranet page. Individuals were selected purposively to represent all levels within the program's decision-making hierarchy; medical and surgical sub-specialties, nursing and quality management; and a range of campuses.

Approach: Personalised email invitations from the project team were sent to the Chair, Executive Sponsor and/or Secretary of 14 committees, managers of 5 APUs and 9 managers from the selected clinical program. Approval from the Nursing and Medical Program Directors was sought before approaching individuals from the selected program.

Interview schedule: Questions were based on the scanning taxonomy (Figure 2). They were piloted with one committee and refined before being used in subsequent interviews. The full interview schedule is available in Table B.

Data collection: Interviews were approximately 1 hour long and were conducted in the interviewee's office or suitable meeting room. Interviews were not taped or transcribed but detailed notes were taken. Two CCE staff members attended, one as interviewer and one as note taker.

Respondent validation: Drafts were sent to the interviewees for clarification, comment and/or amendment as required.

Analysis: Final interview notes were collated and organised in MS Word and Excel using the elements of the scanning taxonomy. Emergent themes were identified by framework analysis.

Response rate: Representatives of 13 of the 14 committees, all 5 APU managers and 9 clinical managers participated. One committee Chair did not respond to the invitation for interview; due to lack of time no representative of this committee was interviewed. A surgical sub-specialty department head was unable to attend their interview and was replaced by a medical sub-specialty department head who was available at short notice.

Representativeness of sample: Almost all eligible committees and all eligible APUs were represented. The clinical managers represented Program Directors, Department Heads, Unit/Ward Managers and ancillary services; medical (n=4), nursing (n=4) and quality management (n=1) staff; in a range of sub-specialties across multiple campuses.

Staff members with experience in disinvestment projects

Aim: To learn from previous experiences of disinvestment at Monash Health.

Inclusion criteria: Staff who had undertaken projects to remove, reduce or restrict current practices (the term 'disinvestment' was not used in Monash Health projects).

Sampling: Purposive and snowball sampling was used. Relevant projects were initially identified by members of the SHARE Steering Committee and interviewees in the committee review process noted above. A snowballing method was employed by asking participating project representatives if they knew of any other relevant projects. Nineteen potential projects were identified, 13 met the inclusion criteria.

Approach: Personalised email invitations from the project team were sent to project managers of 13 relevant projects. Project managers or Department/Unit Heads were sought as key contacts; however a representative of the project team was accepted when a senior staff member was unavailable.

Interview schedule: Questions were designed to explore project governance, use of routinely-collected hospital data, other local data and research evidence in the development and implementation of projects; barriers and enablers to successful project implementation; what staff would do again and what they would do differently. The full interview schedule is available in Table C.

Data collection: Interviews were approximately 1 hour long and were conducted in the interviewee's office or suitable meeting room. Interviews were not taped or transcribed but detailed notes were taken.

Two CCE staff members attended, one as interviewer and one as note taker.

Respondent validation: Drafts were sent to the interviewees for clarification, comment and/or amendment as required.

Analysis: Final interview notes were collated and organised in MS Word and Excel using the elements of the scanning taxonomy. Emergent themes were identified by framework analysis.

Response rate: Representatives of 10 projects participated based on interviewee's and interviewer's availability

Representativeness of sample: The process was designed to be illustrative and did not seek to comprehensively identify all projects. A number of project topics across a range of clinical areas were included.

STRUCTURED WORKSHOPS

SHARE Steering Committee

Aim: The workshops had several aims, those relevant to the research questions in this paper include: To draw on the knowledge and expertise of senior staff to identify systems, processes and people relevant to resource allocation decision-making at Monash Health; to analyse and interpret the findings from these sources; and to make recommendations based on the outcomes.

Inclusion criteria: Senior decision-makers at Executive and Director level and health service consumers

Sampling: Convenience sampling was used to include members of the SHARE Steering Committee comprising Executive Directors (Medical, Nursing, Support Services), clinical Program Directors (Medical, Nursing, Allied Health, Pharmacy, Diagnostic Services), Committee chairs (Technology/Clinical Practice, Therapeutics, Human Research and Ethics, Clinical Ethics), Directors of non-clinical services (Information Services, Clinical Information Services, Procurement, Biomedical Engineering, Research Services), Legal counsel and two consumer representatives. Two representatives from the Department of Human Services Technology Division also participated.

Approach: Workshops were conducted at scheduled Steering Committee meetings.

Design: Workshops were based on the first two steps in the SEAchange model for evidence-based change [1]; identifying the need for change and developing a proposal for change. Presentations outlining the background and aims of the workshops were made by the project team, discussion was structured around the questions to be addressed and decisions were based on consensus. Questions included:

Workshop 1: Where and how are decisions made, documented, communicated, implemented and evaluated and what are the related system issues? Where is change required? Why? What is the problem? How can the need for change be measured? What are the factors enabling sustainability of the current system? How is it integrated?

Workshop 2: What existing systems/processes work well that we could maintain as they are, should be ceased, could be kept but require improvement? What new systems/processes should be introduced? What structures, skills, resources, commitment and leadership are required? Are they available? If not, how can they be obtained? What existing systems can be utilised? What is the solution to the problem? What are the options? What is known about best practice in this area? What is required to ensure sustainability of the proposed system? How can it be integrated?

Data collection: Participants completed prepared worksheets and discussed the findings. Discussion and decisions were documented in minutes.

Respondent validation: Minutes were approved at the following meeting.

Analysis: Data from the worksheets and findings from the discussion were collated and organised in MS Word and Excel. Emergent themes were identified by framework analysis.

Response rate: Thirteen members participated, 9 attended the first workshop, 11 attended the second, and some non-attenders also completed the worksheets.

Representativeness of sample: A range of senior decision-makers were represented at each workshop, plus representatives from the state health department.

Clinical decision-makers from a large diagnostic service

Aim: To capture the actual process of capital equipment purchasing and identify how an ideal process for this decision-making might differ from current practice.

Inclusion criteria: Clinical managers involved in decisions regarding purchase or new or replacement equipment.

Sampling: Purposive sampling was used. A large multi-campus diagnostic service was selected based on their use of equipment and the interest in the project expressed by the Director.

Approach: The Director and Research Director of the department identified 18 suitable participants representing all health professional groups, all campuses and most units within the service. Personalised email invitations were sent by the Executive Director of Medical Services and Quality.

Design: An experienced facilitator from CCE who had no involvement in the SHARE project developed and delivered the workshop. A presentation on the background of the project and its relevance to the workshop was made by a SHARE project team member. Two other project team members were present to assist with logistics and note taking. The session was run over 1½ hours in the departmental seminar room. Five domains were identified a priori: how do we get an idea; what is the process (application, approval, feedback, who, timing); is it a good idea; is it the best idea; and monitoring and evaluation.

Data collection: Using a nominal group technique, participants were asked to describe the ideal process for purchasing large capital equipment. Responses were collected on 'sticky-notes'. This method was

repeated to identify gaps in the current process and included prioritisation of key areas for improvement.<br>**Respondent validation:** A workshop report was provided to participants for comment.<br>**Analysis:** Responses on the 'sticky notes' and additional workshop notes were collated and organised in MS Word and Excel using the domains identified a priori. Emergent themes were identified by framework analysis.<br>**Response rate:** 17 of the 18 invitees attended. An additional staff member from a clinical area not represented on the invitation list was included at the commencement of the workshop.<br>**Representativeness of sample:** Participants represented all campuses, sub-specialties and health professionals (medicine, nursing, allied health, technical, quality improvement, business management, research) within the department.
**DOCUMENT ANALYSIS**
**Aim:** To provide evidence for the stated positions and methods of administration of decision-making systems and processes for resource allocation at Monash Health and the state health department.<br>**Inclusion criteria:** Documents that guided decision-making or implementation of resource allocation decisions<br>**Identification:** Documents were identified by key informants and searches within the Monash Health Policy and Procedure database.<br>**Documents included:** 1) State government: Victorian Government Purchasing Guidelines, Medical Equipment Asset Management Framework, Targeted Equipment Replacement Program and Health Purchasing Victoria Product Management Guidelines. 2) Monash Health: Purchasing Policy, Purchasing Policy Guidelines, Authority Delegation Schedule, Code of Conduct, Conflict of Interest Protocol, Guidelines for management of Gifts and Benefits, Terms of Reference for committees that make resource allocation decisions, Application forms, Business case templates, Requisition forms and checklists.<br>**Data collection:** Documents were retrieved or sourced online. Data were extracted based on the scanning taxonomy.<br>**Analysis:** Findings were collated and organised in MS Word and Excel using the elements of the scanning taxonomy.

Table B. Interview questions for committee decision-making mapped to scanning taxonomy

Characteristics of the external environment (Monash Health) and organisation (Committee)

- What is the role of this committee?
- In what ways do your decisions impact on TCPs?
- Does the committee approve capital expenditure or procurement? And if so, what is the committee's definition of capital?
- Does the committee have a role in developing or approving guidelines or protocols?
- Do any other committees report to this committee?
- Does the committee interact with or refer decisions/applications to other committees?
- Who sits on the committee eg units, departments, professional groups, consumer representation?
- Do committee members have any specific training to sit on this committee? Do you think they require any specific training?
- Are meetings regularly scheduled?
- Is your ability to make decisions affected by attendance?

Characteristics of the potential adopters

- Who would be affected by your decisions?

Characteristics of the innovation (Decision)

- How do issues make the committee's agenda eg application process, referral?
- Does the committee have a conflict of interest procedure for members? For applicants? What is it?
- Are there templates or pro-formas available for applications? Are these easily accessible?
- How are decisions made?
- Are there established, documented criteria for making a decision? If so, are they used?
- Do applicants have to provide evidence for any proposed change? How does the committee judge the quality of the evidence?
- Does the committee use routinely-collected local data eg number of procedures, cost, etc for decision making? Does the committee use data for benchmarking eg department versus department or Monash Health versus other health service?
- What other information or data is considered eg access, equity, legal, financial, etc?
- Does the committee use any priority setting processes in making decisions eg Monash Health strategic plan or DHS initiatives or priorities?
- How are your decisions disseminated? Are minutes or other documents eg decision summaries accessible to non-committee members?
- Is there a process of appeal in dispute of decisions?

Characteristics of the implementation strategy, barriers and enablers

- If a decision is made that changes practice who is responsible for implementing that decision?
- Are support and resources available for implementing decisions?
- Does the committee or Monash Health provide any funding for implementation of major changes?
- Are there any specific barriers or enablers to the committee's work?
- If you have an application process do you think people bypass the system, either deliberately or through lack of knowledge?

Process – degree of implementation

- How do you know if your decisions are being acted upon/followed?
- Is there any evaluation of the committee processes eg user feedback on application forms or resources? Do you have KPIs?

Impact – degree of practice change

- How do you know if your decisions have affected practice?
- How do you monitor and/or evaluate? Do you have KPIs?

Patient outcomes

- Do you collect/measure data about patient outcomes?
- What data are collected/measured and how? Do you collect data on costs to patients? Are existing databases/systems used?
- Who collects the data?

Practitioner outcomes

- Are any outcome data collected from health professionals regarding practice change or satisfaction related to your decisions?

System outcomes

- Can impact be traced to areas other than target areas?

Economic outcomes

- Do you measure any financial outcomes and if so, what?
- Does the committee have sufficient resources to perform its duties?

Reflections

- Is there anything else you want to tell us about your committee?
- Overall, how well do you think the system works?

Snowballing for other interviewees

- Does the committee receive or distribute any alerts from their own research or monitoring or from a third party, eg. TGA recall advice?
- (Other than this list….) Are you aware of any other committees or processes within MH that make decisions that impact on use of TCPs?
- Are you aware of any projects, past or present, within MH that address resource allocation related to new or existing TCPs?

Table C. Interview questions for previous disinvestment projects mapped to scanning taxonomy

Characteristics of the external environment and organisation Please briefly describe the project.

- Tell us a bit about the aims of the project.
- Who initiated the project? (eg Management? Consumer?)
- What are the reasons the project came about? (external influences/drivers related to the project)
  - Internal strategy or priority
  - Funding or resource reasons (internal and external)
  - Responding to patients factors or influences
  - External Policy. Has the project been implemented due to DHS or other government requirements?
- Where does the project fit within the Monash Health reporting structure?

Characteristics of the potential adopters

- Who was the target?
- Why was this group of clinicians/department/behaviour chosen?
- Was any specific training required for the target group?
- Did project staff require education/ training to implement the project?

Characteristics of the innovation

- What type of innovation was implemented? Note: Refer to EPOC definitions
  - Professional
  - Organisational
  - Patient orientated
  - Regulatory
  - Financial (eg funding reliant of results, incentive payment)
  - Structural (eg clinical path)
- Did the project involve the removal of an ineffective, inefficient or unsafe TCP?
- Was there reassessment or restriction of a TCP?
- Was there a reallocation of resources?
- Was your project linked to others that address effective resource allocation?
- Was the project identified through an existing process, such as regular audit, or was it identified independent of such processes (eg just someone's idea)?

Project learnings

- What would you do the same way in future projects? Why?
- What would you do differently? Why?

Other questions as per committee decision-making interview schedule

Table D. SHARE Steering Committee Workshop Proformas

WORKSHOP ONE

Presentation and Discussion

Background

Step 1. Identify the need for change

Where is change required? Why? What is the problem? How can the need for change be measured? What are the factors enabling sustainability of the current system? How is it integrated?

Worksheet questions

Section 1: Consider decision-making for Capital Procurement and Clinical Purchasing (expenditure) and Guidelines and Protocols (allocation of non-monetary resources)

Where are decisions made?	How are decisions made?	How are decisions documented?	How are decisions communicated/implemented?	How are decisions evaluated?	What are the relevant system issues?	Contact person/s
Eg Standing Committees	Are there explicit decision-making criteria? Is there explicit use of evidence (research literature or local data)? Is there a priority setting process? Is there an application process?	How are decisions documented? Are minutes accessible to non-committee members? (or something similar such as a decision summary or other documentation?)	How are decisions disseminated?<br>Who is responsible for implementing that decision? Are support/resources available for implementing decisions?	How do you know if your decisions are being acted upon/followed? Is there any evaluation of the committee processes? Are any outcome data collected?	What structures, skills, resources, leadership and commitments are involved currently? What communication systems are in place? How well does this integrate with other MH processes?	People who could provide additional information

Section 2: System-wide or Specific (examples from Section 1 or other settings)

Decision-making setting	What works well?	What doesn't work well?	How can we improve it?	Where are the gaps?	What can we learn from current or previous work?	Contact person/s

WORKSHOP TWO

Presentation and Discussion

Findings from Workshop One

Step 2. Develop a proposal for change

What is the solution to the problem? What are the options? What is known about best practice in this area? What is required to ensure sustainability of the proposed system? How can it be integrated?

Worksheet questions

	System or process	Details/thoughts	What structures, skills, resources, commitment and leadership are required? Are they available? If not, how can they be obtained?<br>What existing systems can be utilised?	Contact person/s
What existing systems/processes work well that we could maintain as they are?				
What existing systems/processes should be ceased?				
What existing systems/processes could be kept but require improvement?				
What new systems/processes should be introduced?				

Abbreviations

APU	Approved Purchasing Unit
CCE	Centre for Clinical Effectiveness
DHS	Department of Human Services
MH	Monash Health
SHARE	Sustainability in Health care by Allocating Resources Effectively
TCP	Technology and clinical practice
TGA	Therapeutic Goods Administration

References

1. Harris C, Turner T, Wilkinson F. SEAchange: Guide to a pragmatic evidence-based approach to Sustainable, Effective and Appropriate change in health services. 2015. Available from: http://arrow.monash.edu.au/hdl/1959.1/1225377. Accessed: October 2016
