# Supplementary material for: Sustainability in health care by allocating resources effectively (SHARE) 3: examining how resource allocation decisions are made, implemented and evaluated in a local healthcare setting
Source: BMC Health Serv Res. 2017 May 9;17:340. doi: 10.1186/s12913-017-2207-2 (PMC5423420; doi:10.1186/s12913-017-2207-2)
Supplement: Supplementary file 2 — Strengths and weaknesses, barriers and enablers for resource allocation processes (PDF 463 kb) [file 12913_2017_2207_MOESM2_ESM.pdf]

The SHARE Program (Sustainability in Health care by Allocating Resources Effectively) 3: Examining how resource allocation decisions are made, implemented and evaluated in a local healthcare setting

Additional File 2

Strengths and weaknesses, barriers and enablers for resource allocation processes

Contents

External environment 2
- General 2
- International 2
- National 2
- State 2

Monash Health environment: General 2
Monash Health environment: Governance 3
- Oversight 3
- Policies and procedures 3
- Transparency and accountability 3
- Conflict of interest 4
- Monitoring, evaluation and improvement of systems and processes 4
- Reporting 4

Monash Health environment: Administration 4
- Relationships, coordination, collaboration and communication 4

Monash Health environment: Stakeholder engagement 4
Monash Health environment: Resources 5
- Funding and staff time 5
- Expertise and Training 5
- Information 5

Decision-makers 6
Potential adopters 6
Decision-making process 6
- Identification of need/application 6
- Decision criteria 6
- Ascertainment and use of evidence 6
- Reminders and prompts to consider disinvestment 7
- Deliberative process 7
- Documentation and dissemination 7

Implementation 7
- Purchasing 7
- Policy and guidance 8
- Implementers 8
- Practice change 8

Evaluation of outcomes of decisions 9
- General 9
- Evaluators 9
- Requirements for evaluation 9

Reinvestment 9

Strengths and weaknesses in decision-making for resource allocation

Factors identified in response to a specific question about barriers and enablers are noted in italics

STRENGTHS	WEAKNESSES
**External environment**	
**General**<br>▪ *Good relationships with external agencies such as Australian Council of Healthcare Standards, Victorian Department of Human Services (DHS)*<br>▪ *Projects initiated by external organisations such as Australian Quality Council, NSW Therapeutics Advisory Group and Clinical Excellence Commission*	
▪ Legislation, regulations, national and international standards, and professional standards must be followed. This provides clarity and certainty for some decisions.	▪ Some decision-makers are unaware of mandatory requirements.
**International**<br>▪ International bodies and national agencies of other countries provide evidence-based recommendations for use of health technologies, clinical practices, models of care, etc.<br>▪ Systematic reviews and Health Technology Assessments are also available.	▪ Decision-makers are frequently unaware of these resources.<br>▪ Due to lack of time, knowledge and skills decision-makers do not actively seek these resources when making decisions and do not differentiate between high and low quality resources.<br>▪ Cost-effectiveness data is often based on modelling which is perceived not to reflect reality
**National**<br>▪ The Medical Services Advisory Committee and Pharmaceutical Benefits Advisory Committee provide evidence-based recommendations for use of medical and surgical procedures and drugs.	▪ Not all medical and surgical procedures and drugs are covered by these processes.<br>▪ Nursing and allied health practices, models of care and clinical consumables are not covered.
**State**<br>▪ Guidance for introduction of new health technologies and clinical practices (TCPs) is provided by DHS. This includes reporting requirements.<br>▪ Monash Health has developed tools to implement these processes. DHS has recommended these tools to other health services.<br>▪ Monash Health Decision Summaries are published on the health service website.	▪ *DHS requirements and processes are cumbersome*<br>▪ There is no sharing of information or decisions. Individual health services duplicate the process of finding and appraising relevant evidence, developing business cases, etc.<br>▪ DHS declined to coordinate sharing of information through a central database or website.
▪ The Victorian Policy Advisory Committee on Technology (VPACT) has an annual funding round for introduction of new high cost TCPs	▪ Respondents unaware of any long term state-wide strategic planning for equipment purchases<br>▪ Lack of coordination of equipment use and procurement at state level and no communication between health networks.
▪ Some guidance for purchasing is provided through the Victorian Government Purchasing Guidelines, Medical Equipment Asset Management Framework (MEAMF), Targeted Equipment Replacement Program (TERP) and Health Purchasing Victoria (HPV).<br>▪ HPV is responsible for bulk purchasing of pharmaceuticals, clinical equipment and consumables to streamline ordering and reduce costs. If the item required is in the HPV catalogue the specified brand must be purchased from the designated suppliers at the cost and conditions noted.<br>▪ The processes are transparent and accountability is clear.	▪ HPV catalogue only covers 30% of Monash Health consumables<br>▪ Inclusion of items in the HPV catalogue is not always based on a rigorous evidence-based process<br>▪ Safer, more effective or more cost-effective alternatives may not be included in the catalogue<br>▪ HPV does not cover large items so MEAMF and TERP have no benefits from bulk purchasing and hospitals have to negotiate their own arrangements with suppliers<br>▪ Decision-makers do not know which of these multiple systems are relevant to a particular situation<br>▪ Terminology differs between systems and they are difficult to navigate
▪ The Victorian Aids and Equipment Program is administered by Monash Health on behalf of the DHS. The application process is standardised based on tight explicit criteria for transparency and accountability.	▪ This is a 'last resort' process after other sources of funding have been exhausted. Clinicians waste valuable time writing funding applications for multiple programs which could be integrated and allocated centrally.
▪ The Department of Treasury is interested in supporting disinvestment initiatives but requires details of savings. If savings or reinvestments can be quantified the department may provide more funding.	▪ It is hard to measure the savings<br>▪ The savings are rarely realised because they are absorbed and used to treat more patients
**Monash Health environment: General**	
▪ *Enthusiastic and dedicated staff*<br>▪ *Staff commitment to quality improvement*	▪ *High staff turnover in the organisation, particularly agency nurses and junior staff, increases difficulty in communication and implementation*

▪ *Organisational support*<br>▪ *Support from the Executive Management Team*<br>▪ *Support from Directors of Nursing*<br>▪ *Involvement of people who are outside of, or uninterested in, the politics of the organisation*	▪ *High staff turnover in projects diminishes organisational knowledge and expertise and increases training requirements*<br>▪ *Organisational culture is difficult to change*<br>▪ *Organisational politics*<br>▪ *Incident reporting software (Riskman) is flawed, does not cover all requirements and does not enable valid aggregation of data related to consumer information*
▪ Strategic planning provides an opportunity for integrating disinvestment decisions into organisational practices. Monash Health had transparent strategic and business planning processes	▪ Lack of strategic planning for large equipment purchases
▪ The Board, Executive Management Team (EMT) and Senior Managers have expressed 'patient-centred care' as a priority.	▪ Considerable pressures on the health service to reduce costs.<br>▪ Perceived distinction between 'what the hospital is concerned about (finances, organisational capacity and risk management) and what the clinician is concerned about (patients)'.
**Monash Health environment: Governance**	
**Oversight**<br>▪ Overall accountability sat with the Monash Health Board. The Board and EMT determined the decision-making structures within the organisation.<br>▪ The Quality Unit maintained an organisational chart of committees related to quality and safety.<br>▪ The Board Secretary also had a list of some committees	▪ No central resource for oversight, coordination or provision of information about committee processes<br>▪ No complete list of committees operating at an organisation-wide level<br>▪ No lists of committees operating within programs or sites
**Policies and procedures**<br>▪ *Robust policies and guidelines for purchasing*<br>▪ *Relevant Terms of Reference for committees*	
▪ Nature and scope of decisions was stipulated in the Purchasing Policy, Purchasing Policy Guidelines and Authority Delegation Schedule to prevent gaps, overlap and ambiguity.	▪ Confusion about 'who does what'<br>▪ Duplication of some committee and project activities
▪ In addition to policies and guidelines there were supporting documents such as application forms, business case templates, requisition forms and checklists governing activities related to resource allocation such as purchasing and procurement and development of clinical guidance documents.	▪ *Too much paperwork and existing paperwork is confusing and ambiguous*<br>▪ Some documents were not well organised, not easily accessible, multiple versions were available and some required considerable skills and resources to complete<br>▪ Emphasis on 'business' aspects and less consideration of evidence of safety, effectiveness and cost-effectiveness in many of these documents
**Transparency and accountability**<br>▪ Transparency and accountability in decision-making was highly valued by respondents<br>▪ Improved transparency and accountability at Monash Health was desired by most respondents	▪ *Lack of transparency in all aspects*<br>▪ *Lack of transparency and accountability in decision-making reduces confidence*<br>▪ Inadequate transparency and accountability was one of the strongest messages from respondents
▪ Clear documented lines of accountability and reporting requirements in some areas<br>▪ Individuals and members of committees at the top of their respective decision-making hierarchies reported that they had clear understanding of how the processes should work, who is accountable, who makes the decision, etc and knew the difference between recommendations, decisions and authorisation.<br>▪ Many of these respondents also reported that all decision-makers have the same understanding as they do.	▪ Many individual and group decision-makers lower down the respective hierarchies admitted they were unsure of the processes. Others who said they were sure gave answers that were inconsistent with each other. Some reported ambiguities and inconsistencies in the systems and processes.<br>▪ Confusion between the concepts of 'decision' and 'recommendation' which may lead to uncertainty in accountability. Some committees saw their role as 'recommending' a course of action with the 'decision' being made by a higher level committee. In contrast, the higher level committees saw their role as one of guidance and support in response to robust investigation of decision options which they expected to occur at the lower level 'decision-making' committees.<br>▪ Individual decision-makers did not always know who to report a decision to and whether formal authorisation was required.

**Conflict of interest**<br>▪ Conflict of Interest required as a standing item on the agendas of relevant committees. Ten of 13 committees interviewed had a process for conflict of interest for committee members, and two of the four committees with an application process had a similar procedure for applicants.	▪ Only one committee, the Technology/Clinical Practice Committee (TCPC), considered the effect of conflict of interest in the provision of evidence used in decision-making
**Monitoring, evaluation and improvement of systems and processes**<br>▪ Quality improvement of systems and processes was supported by respondents<br>▪ Only one committee (TCPC) had an ongoing process of monitoring, evaluation and improvement of its systems and processes, however some committees had undergone a single evaluation/review and some were developing or planning to develop quality improvement processes.	▪ No formal requirements for quality improvement of decision-making at Monash Health<br>▪ At the program level it was noted that 'since there was no formal decision-making process there was no process of review'.
▪ Committees that authorise or support decisions made by other committees expected that a rigorous process of decision-making and prioritisation had occurred	▪ No system to check or regulate this
**Reporting**<br>▪ Quality Unit chart of committees related to quality and safety included lines of reporting<br>▪ Most committees had reporting requirements included in their Terms of Reference	▪ The structure and process of reporting varied with site, department/unit and health professional group making the decisions across and between sites, programs, units, etc difficult<br>▪ No systematic or documented process for reporting of projects
**Monash Health environment: Administration**	
**Relationships, coordination, collaboration and communication**<br>▪ *Knowing who to go to for information*<br>▪ *Knowing who to go to for support*<br>▪ *Networks within the organisation, particularly nursing*<br>▪ *Quality and Risk Managers are good at sharing information across the organisation*<br>▪ *Good communication at site level (nursing)*<br>▪ *Robust and regular communication*	▪ *Lack of knowledge and awareness about*<br>· *decision-making systems and processes and where to go to find out about them*<br>· *information sources and tools and where to go to find them*<br>▪ *Lack of information regarding how the system works and what processes need to be followed*<br>▪ *Lack of central resource/identified role to provide information about committees*<br>▪ *Lack of organisational processes for knowledge transfer*<br>▪ *Lack of coordination and collaboration between decision-making individuals and groups*<br>▪ *Lack of communication about decisions between programs, departments and other stakeholders*<br>▪ *Lack of communication about impending decisions and projects to enable stakeholder input*
▪ Quality Unit chart of committees included relationships (but only for reporting purposes).<br>▪ Some committees recognised the overlap in their work and the potential to work together. These were in two groups, those considering introduction of new TCPs and those involved in purchasing.<br>▪ People who were members of more than one committee often provided the links between them.<br>▪ There were many examples of cross-unit/department consultation and collaboration for policy and protocol development and implementation.<br>▪ Four projects were linked to others with similar aims	▪ Lack of awareness of other committees within Monash Health<br>▪ Other than reporting, there were no documented relationships between committees<br>▪ Other than the committees considering new TCPs, there were no formal processes of referral for issues that might affect, or should be addressed by, other committees<br>▪ Decision-making 'in isolation' was noted to be a problem in multiple settings. 'Fragmentation' and a 'silo mentality' were used in relation to decisions made without consideration of the areas they will impact upon or consultation with relevant stakeholders.<br>▪ No systematic processes to link projects across the organisation
**Monash Health environment: Stakeholder engagement**	
▪ *Involvement of broad range of stakeholders from multiple sites and a range of health professional disciplines*<br>▪ Reported benefits of broad stakeholder involvement in decision-making included improved decision-making, more effective dissemination of decisions and informing and encouraging others about the need to consult with the groups represented	▪ *Lack of consultation with clinicians in decisions made by managers*<br>▪ *Lack of consideration of impact of change on others when making decisions or planning projects*<br>▪ *Lack of consideration of downstream or lateral impacts eg 'cost saving measures in one area can result in increased costs in another area'*<br>▪ *Limited input from the Quality and the Education Units*
▪ Many respondents supported increased consumer participation and were planning to act upon this	▪ Only one committee (TCPC) included consumer representation in decision-making.<br>▪ Several respondents thought that consumer representation on their committees would be inappropriate or that consumers had insufficient technical understanding to participate.

Monash Health environment: Resources	
**Funding and staff time**<br>▪ *Provision of extra staff*<br>▪ *Availability of extra funds enhanced implementation and evaluation, eg introduction of the National Inpatients Medication Chart had external funding specifically for implementation and evaluation*<br>▪ *Some clinical pathways involve no additional costs*	▪ *Lack of/inadequate funding resulted in*<br>· *lack of/inadequate administration*<br>· *lack of/inadequate evaluation and research*<br>· *compromised building cost estimates, hindering capacity to house/operate equipment properly*<br>▪ *Funding for new equipment frequently did not include funding for training staff to use it or the consumables required.*<br>▪ *Lack of information about available funding*<br>▪ *Staff dissatisfaction with the expectation of their superiors that they will do more work within existing resources*<br>▪ *Insufficient allocation of staff time impairs*<br>· *research and preparation for decisions*<br>· *implementation and evaluation of decisions*<br>· *project delivery*<br>· *training*<br>▪ *Lack of/inadequate coordination of current resources*
▪ Some committees had a Secretariat comprised of 1-2 officers from named roles within the organisation. These positions were allocated sufficient time to complete the required tasks.<br>▪ Some projects were provided with adequate resources for implementation and evaluation<br>▪ Some wards had additional staffing for education support and clinical nurse support. These were invaluable resources for practice change, protocol development and implementation.<br>▪ Some projects had external funding from DHS, universities, etc for staff or infrastructure costs	▪ Some committees used the Personal Assistant of the committee Chair in an administrative role. If a new Chair did not have a personal assistant there would be no resources to support the committee.<br>▪ Some respondents found it difficult to separate the role of the committee from the role of their department. Committee work significantly increased their overall workload, particularly administrative matters, and it was not always clear if these duties were part of, or additional to, their normal duties and what they could cut back in order to accommodate committee obligations.<br>▪ Many projects were to be carried out 'within existing resources'. Respondents noted that they either did unpaid overtime or aspects of the project were not undertaken.
**Expertise and Training**	▪ *Lack of/inadequate skills in*<br>· *use of information technology*<br>· *finding and appraising evidence from research and data*<br>· *project management*<br>· *change management*
▪ Staff in Centre for Clinical Effectiveness (CCE) and Clinical Information Management (CIM) were available to decision-makers to provide expertise in research evidence and local data respectively.<br>▪ CCE ran training programs in finding and using evidence, implementation and evaluation<br>▪ Six of 10 projects had training for project staff in change management, leadership or IT skills.	▪ CCE's funding for training was redirected due to budget cuts so it was unable to provide free in-house programs (however many staff attended the fee-paying courses CCE provided)<br>▪ Lack of understanding of information systems and project management in senior decision-makers was reported and training for committee members was suggested<br>▪ Most projects used a staff member from the department involved to deliver the project, most of these did not have project skills or expertise.<br>▪ Education and training is not well provided for part-time and night staff
**Information**<br>▪ *Provision of extra computers*	▪ *Lack of computers and/or access to computers, particularly for nurses*<br>▪ *Difficulties using intranet to find organisational data*
▪ CCE and CIM were available to provide information to decision-makers<br>▪ Monash Health libraries provided access to health databases and electronic journals, as well as advice in searching the health literature	▪ *Lack of research evidence and local data to inform decisions*<br>▪ Many decision-makers chose not to use these sources of information<br>▪ Priority was given to senior decision-makers and high level decisions; sometimes decisions at lower levels could not be provided with information due to limited resources

**Decision-makers**	
▪ *Broad committee membership*<br>▪ *Dedication of committee members*<br>▪ *Depth and range of experience of committee members*<br>▪ *Proactive clinicians who think about improving and moving forward*<br>▪ *High level of skill within medical staff acting as leaders in their specialties*	▪ *Clinical autonomy*<br>▪ *High workload in running a committee with lack of administrative staff*<br>▪ *Difficulty taking off 'clinician hat' and replacing it with 'manager or decision-maker hat'*
▪ Committee membership included a range of relevant stakeholders (except consumers) invited to participate because of their role in the organisation or their knowledge and skills in relevant areas.	▪ *Some clinicians feel that if they are experts in a particular area they should not have to justify operational decisions*
**Potential adopters**	
▪ *Having the appropriate profession engaging others in change process, for example nurses should be implementing projects with nurses, not pharmacists.*	▪ *Resistance to change*<br>▪ *Staff cynicism about the importance of changes and relevance to them*<br>▪ *Some clinicians insist on autonomy in their areas of expertise*
**Decision-making process**	
**Identification of need/application**<br>▪ Decisions were instigated by 'top down' direction and 'bottom up' invitation.	▪ General perceptions that<br>· financial drivers were stronger than clinical drivers<br>· impetus for change was ad hoc, there was no systematic or proactive approach<br>· internal bureaucracy and red tape stifled ideas
▪ Some committees had a well-documented application process.	▪ *Complex and time consuming nature of application processes*<br>▪ *People by-pass the system, usually not deliberate but due to lack of awareness of the process*<br>▪ Some applications are driven by pharmaceutical or equipment manufacturers
**Decision criteria**<br>▪ Documenting explicit criteria was generally viewed positively.<br>▪ The committees with application forms had some documentation of criteria.<br>▪ Other decision-making groups and individuals had 'mental checklists' of criteria they considered.	▪ Only one committee (TCPC) and one individual used explicit, documented decision-making criteria.<br>▪ Some committees had no decision-making criteria.<br>▪ Some individual decision-makers strongly rejected documentation of explicit criteria as 'another form of paperwork that will waste clinician's time'.
▪ Most committees considered the Monash Health Strategic Plan, quality, safety, access and equity.<br>▪ All committees considered financial factors.	▪ *Organisational priorities dominated eg*<br>· *'Sound practice is not always affordable practice'*<br>· *'The operational aspects of nursing (Key performance indicators that are reported to DHS) come first and professional aspects comes second'*<br>▪ There was a perception that there was 'too much emphasis on financial return for investment'
**Ascertainment and use of evidence**<br>▪ *Strong knowledge of the literature*<br>▪ *Attendance at conferences*<br>▪ Using research evidence and local data in decision making was considered to be important.<br>▪ All respondents reported using research evidence and data in decision-making to some extent.<br>▪ Most committees sought a broad membership in order to utilise expertise in the consideration of research evidence and for decision-making with limited evidence.<br>▪ Four out of ten projects sought research evidence from the literature to inform the project.	▪ *Amount of time needed to search the literature or collect data*<br>▪ *Access to evidence is not easy or coordinated*<br>▪ *Lag time between what universities teach and latest research evidence so new staff are not always aware of best practice*<br>▪ *Drug company marketing*<br>▪ Only one committee (TCPC) required explicit inclusion of research and local data and considered the quality and applicability of this evidence. Only one of the projects appraised the evidence used.<br>▪ The other committees had no process to seek evidence from research. When evidence from research and data was used it was not usually appraised for quality or applicability.<br>▪ Due to difficulty finding uninterrupted blocks of time, slow computers and lack of skills in finding and analysing evidence, decision-makers relied on clinical expertise and advice from colleagues.<br>▪ Appropriate local data was frequently reported to be lacking, unavailable and 'manipulated'.

**Reminders and prompts to consider disinvestment**<br>▪ One application form (TCPC) had an explicit question about what the new technology will replace and what can be disinvested.	▪ 'It's all very well to ask the question but it's very hard to get a clinician to say they will stop doing something'.
**Deliberative process**<br>▪ *Robust and honest conversations*<br>▪ *Autonomous decision-making*<br>▪ Decision-makers expressed a desire for a documented standard process.<br>▪ Many respondents noted that the main goal of discussion was to reach decisions by consensus.	▪ *Process not seen as priority for some*<br>▪ *Some committee members do not attend*<br>▪ *Meetings too short for proper deliberation*<br>▪ *Some decisions made reactively, 'on the run', due to lack of consultation or not following process*<br>▪ *Long lag time between application and decision*<br>▪ Lack of standardised process<br>▪ Many of the current processes were perceived to be unclear, 'ad hoc' and lacking objectivity<br>▪ Lobbying, both covert 'behind the scenes' and overt 'squeaky wheels', was perceived to result in favourable decisions.
▪ Most committees required not only the presence of a quorum to make decisions but also attendance of members with relevant knowledge or expertise to the decision at hand	▪ Not all committees had a defined quorum. Of those that did, some made decisions in the absence of a quorum and some made decisions even if a meeting was cancelled due to lack of a quorum<br>▪ Some decisions were made outside committee meetings or by the Chair only
**Documentation and dissemination**<br>▪ One committee (TCPC) published Decision Summaries which were formally distributed to the Therapeutics Committee, EMT, DHS, the Applicant, Department Head and Program Head and made publicly available on the internet.<br>▪ Most committees recorded minutes; these were considered to be confidential and were not published, but were available to appropriate requestors by contacting the committee secretariat<br>▪ All of the individual decision-makers interviewed reported disseminating decisions to people they considered appropriate and, when deemed necessary, disseminating decisions organisation-wide.<br>▪ Many respondents reported others disseminating decisions to them.	▪ *Large size, nature and diversity of the organisation increases*<br>▪ *difficulty in dissemination of information*<br>▪ *frequency and range of communication methods required*<br>▪ *Not everyone uses email*<br>▪ *Using email too often dilutes the effect*<br>▪ The majority of committees did not publish minutes or anything similar.<br>▪ One committee did not keep any records.<br>▪ Although some related committees exchanged minutes there was a lack of formal communication across committees.<br>▪ Documentation and dissemination of decisions made by individuals was informal and ad hoc.<br>▪ Not all projects communicated decisions to other staff members or the wider organisation. Unless people were directly involved, some projects appeared not to make project work or associated decisions public knowledge.<br>▪ Lack of processes for knowledge transfer, especially across sites.
**Implementation**	
**Purchasing**<br>▪ Robust organisational processes that met annual audit requirements<br>▪ Electronic ordering was controlled through an approval hierarchy with delegation thresholds.<br>▪ It was assumed that the decision to purchase was made with due process before reaching the purchasing unit.	▪ Use of evidence in purchasing decisions was not outlined in the Purchasing Policy Guidelines.<br>▪ Those making the decision of 'whether to buy' were responsible for ascertaining evidence of safety, effectiveness and cost-effectiveness in the first stage; however there was no system to check that this has been done before the second stage.
▪ Health Technology Services, the Product Evaluation Committee and working parties set up to evaluate large individual capital purchases considered appropriateness of equipment to Monash Health, availability of spare parts, life expectancy, servicing requirements, related consumables, availability of technical expertise and fit with the DHS Asset Management Framework. They also had expertise in contract negotiation.	▪ *Difficulty managing expectations eg 'once something is approved people want it immediately'*<br>▪ Some were unaware of this process and went directly to the manufacturer. If this was overseas it may be difficult or expensive to get parts, there may not be relevant skills for local maintenance and it excludes benefits that may already exist with a local manufacturer that could supply the same product under better terms and conditions. Re-negotiating contracts, or establishing new ones, creates bad feeling and wastes lots of time.

▪ Purchasing of clinical consumables within budget allocation is done electronically. Electronic authorisation is required for items above individual limits (eg Nurse Unit Manager approval up to $10,000, items above this require authorisation)	▪ There is little assessment of safety, effectiveness or cost-effectiveness of clinical consumable items
**Policy and guidance**<br>▪ Monash Health was developing a new Policy and Procedure Framework<br>▪ Broad support for increased standardisation of practice through policies and procedures<br>▪ Development process seen as a communication tool between professional groups and across sites	▪ Lack of structure and standardisation of processes, especially between sites
**Implementers**<br>▪ *Finding others who have done the same work for support, advice and information*<br>▪ *Establishing Working Parties and Steering Committees for support, endorsement, troubleshooting*<br>▪ *Project leader whose primary role is 'at the coal face'*<br>▪ Decisions made at program level that involve multiple wards, departments or sites are usually implemented by multidisciplinary teams	▪ Some project staff felt isolated and would have liked support from others who had done the same or similar work<br>▪ It was not always clear who was responsible for project management<br>▪ *Lack of/inadequate project management and communication resulted in multiple people*<br>· *making inconsistent changes*<br>· *contacting equipment vendors with requests and ideas for change*
**Practice change**<br>▪ *At site level there is good 'buy-in' for change and people are keen to make things work (nursing)*<br>▪ *Allowing wards to nominate themselves for participation in projects*<br>▪ *'Bottom up' approach to develop individual implementation plan in each ward*<br>▪ *'Bottom up' training to gain staff 'buy in' combined with 'top down' supportive strategy*<br>▪ *Flexible and adaptable staff*<br>▪ *Lots of preparation including training and communication with all stakeholders*<br>▪ *Use of pre-existing (and pre-tested) tools from other organisations*	▪ *Unrealistic project timelines*<br>▪ *Variability in current practice and lack of standardisation increases number of practices to change*<br>▪ *Large size, nature and diversity of the organisation increases complexity of implementation across departments with different needs*<br>▪ *Lack of effective implementation pathways*<br>▪ *Things take a long time to implement, to the point that they 'fall off the agenda'*<br>▪ *Staffing issues, including leave, mean that a lot of projects are on hold*<br>▪ *Project-specific barriers such as logistical challenges with product being implemented*
▪ Some committees provide an approval process only and the applicant is responsible for implementing the decision. In most cases the applicant has control over the process (eg head of department implementing a new procedure) and is motivated to implement the change	▪ Sometimes practice change is required beyond the applicant and their department. Committees do not require applicants to have or acquire knowledge and skills in implementation.
▪ Training and education activities and 'champions' were reported as the two key strategies used to effect change and encourage sustainability of the intervention.<br>▪ Most projects had a champion and/or Executive sponsor. Project champions were generally the head of the relevant department; others included the Chief Executive Officer, Executive Directors who were Steering Committee Chairs and 'Ward Champions' selected to encourage and promote change.<br>▪ Those with champions unanimously considered champions important to the success of the project.<br>▪ Training or education included passive methods using posters and memos, interactive learning on new equipment and participatory approaches involving staff in design and implementation.<br>▪ Seven projects involved training for the target group, most of which was done by external providers of new equipment.	▪ Lack of knowledge and skills in project management, change management and use of information technology were exacerbated when interventions were complex and required high levels of training<br>▪ Lack of known, standardised processes for implementation at Monash Health
▪ Most considered their project sustainable and believed the change was embedded in the system. This was reportedly achieved by involving a variety of staff and 'bottom-up' approaches to change.	▪ Only two considered sustainability in the design of the project.
▪ Half of the projects tailored the implementation plan to anticipated barriers and enablers sourced from other health services, literature searches and personal experiences of project staff.<br>▪ Half reported that implementation was conducted as planned. Some noted that it mostly went to plan but 'amendments were made continually to improve the process'.	▪ One project had no implementation plan<br>▪ Half of the projects did not consider barriers and enablers
▪ *The benefit of the proposed practice change is clear and observable*	▪ Lack of baseline data meant that potential adopters were unable to see the benefit or relevance to their situation resulting in less 'buy in' and poor uptake.

**Evaluation of outcomes of decisions**	
**General**<br>▪ *Use of pre-existing (and pre-tested) tools from other organisations eg audit tools*<br>▪ Evaluation and monitoring were considered important and had broad support<br>▪ Monitoring of projects after implementation was thought to increase sustainability	▪ *Quality and Risk Managers are not included at the beginning to help with collection of baseline data and evaluation design*<br>▪ Lack of baseline data<br>▪ A lack of data was seen to contribute to the current state of 'little or no process of evaluation'.<br>▪ Limited funds, knowledge and/or skills inhibited both the planning and conduct of evaluation.
**Evaluators**<br>▪ CCE was establishing an in-house Evaluation Service at the time of these interviews.	▪ No specified evaluators with appropriate training or expertise had been utilised by the respondents
**Requirements for evaluation**<br>▪ Monitoring, evaluation and reporting of outcomes was required by DHS sponsored projects and TCPC. The Therapeutics Committee requested reports for some decisions.<br>▪ Routine clinical audits and monitoring of adverse events undertaken for hospital accreditation purposes provided indirect evaluation of decisions in some situations.<br>▪ Half of the completed projects had been evaluated; all but one project reported achieving its planned objectives.	▪ Monash Health had no requirements for evaluation of outcomes of decisions or projects.<br>▪ Most committees had no planned evaluation of outcomes of decisions or implementation projects.<br>▪ The purpose of reports for TCPC and Therapeutics was questioned by some respondents who noted that it may be inconsistent with the knowledge needed for program staff.<br>▪ Only 2 projects planned evaluation as a project component. Some were evaluated post hoc.
**Reinvestment**	
▪ *Reinvestment or reallocation of resources would be an incentive to disinvestment*<br>▪ SHARE Steering Committee keen to establish and support methods for reinvestment/reallocation<br>▪ Flexibility and thinking laterally to include novel methods/indicators such as reducing waiting lists, getting patients out of Emergency Department faster, freeing up time in procedural/operating suites, freeing up bed days that are used to treat another patient group faster (eg X procedure saved Y$/bed days which was used by Z patients).	▪ *Lack of planning for resource reallocation*<br>▪ *Lack of transparency and consultation in reallocation of savings creates disillusionment*<br>▪ *Staff dissatisfaction that savings generated are not reallocated*<br>▪ A health economist is required to do this properly, Monash Health had no resources for this<br>▪ 'We don't look far enough for downstream effects; we're too simplistic in assessment of savings'.<br>▪ It was noted that savings made in a project in one area sometimes increased costs in other areas; hence reallocation of the savings to the project department would be unfair.<br>▪ Savings of bed days or time in procedural/operating suites were used immediately to treat another patient group so were never realised<br>▪ Accounting practices did not enable measurement and/or reallocation of savings in some areas, for example changes to one TCP may affect multiple cost centres eg department, ward, ICU, pharmacy
